# Quercetin Protects Blood–Brain Barrier Integrity via the PI3K/Akt/Erk Signaling Pathway in a Mouse Model of Meningitis Induced by *Glaesserella parasuis*

**DOI:** 10.3390/biom14060696

**Published:** 2024-06-14

**Authors:** Peiyan Sun, Yaqiong Yang, Linrong Yang, Yuanzhuo Qian, Mingxia Liang, Hongbo Chen, Jing Zhang, Yinsheng Qiu, Ling Guo, Shulin Fu

**Affiliations:** 1 Laboratory of Genetic Breeding, Reproduction and Precision Livestock Farming, School of Animal Science and Nutritional Engineering, Wuhan Polytechnic University, Wuhan 430023, China; 2 Hubei Key Laboratory of Animal Nutrition and Feed Science, Wuhan Polytechnic University, Wuhan 430023, China; 3 Hubei Collaborative Innovation Center for Animal Nutrition and Feed Safety, Wuhan 430023, China; 4 Hubei Provincial Center of Technology Innovation for Domestic Animal Breeding, Wuhan 430023, China

**Keywords:** *Glaesserella parasuis*, quercetin, inflammation, blood–brain barrier, tight junction, brain microvascular endothelial cells

## Abstract

*Glaesserella parasuis* (*G. parasuis*) causes serious inflammation and meningitis in piglets. Quercetin has anti-inflammatory and anti-bacterial activities; however, whether quercetin can alleviate brain inflammation and provide protective effects during *G. parasuis* infection has not been studied. Here, we established a mouse model of *G. parasuis* infection in vivo and in vitro to investigate transcriptome changes in the mouse cerebrum and determine the protective effects of quercetin on brain inflammation and blood–brain barrier (BBB) integrity during *G. parasuis* infection. The results showed that *G. parasuis* induced brain inflammation, destroyed BBB integrity, and suppressed PI3K/Akt/Erk signaling-pathway activation in mice. Quercetin decreased the expression of inflammatory cytokines (*Il-18*, *Il-6*, *Il-8*, and *Tnf-α*) and BBB-permeability marker genes (*Mmp9*, *Vegf*, *Ang-2*, and *Et-1*), increased the expression of angiogenetic genes (*Sema4D* and *PlexinB1*), reduced *G. parasuis*-induced tight junction disruption, and reactivated *G. parasuis*-induced suppression of the PI3K/Akt/Erk signaling pathway in vitro. Thus, we concluded that quercetin may protect BBB integrity via the PI3K/Akt/Erk signaling pathway during *G. parasuis* infection. This was the first attempt to explore the protective effects of quercetin on brain inflammation and BBB integrity in a *G. parasuis*-infected mouse model. Our findings indicated that quercetin is a promising natural agent for the prevention and treatment of *G. parasuis* infection.

## 1. Introduction

*Glaesserella parasuis* (*G. parasuis*, GPS), which causes meningitis, polyserositis, arthritis, and frequently pneumonia-like symptoms, is the etiological agent of Glässer’s disease in pigs [1]. Meningitis is the most characterized symptom of this disease [2]. Infection with *G. parasuis* always results in high morbidity and mortality and leads to significant economic losses for the pig industry [3]. Despite this, the pathogenesis of *G. parasuis* remains largely unknown. Fifteen serovars have been identified, and numerous serovars do not induce protective cross-immunity [4]. Antimicrobials are commonly used to control *G. parasuis* infection, but their effects are suboptimal [5]. As the problem of bacterial resistance has become increasingly serious, it has become essential to identify the pathogenesis and explore new alternative substitutes for preventing and controlling *G. parasuis* infection.

Meningitis, a typical clinical symptom of *G. parasuis* infection, is defined as inflammation of the meninges [6]. The pathological process of bacterial meningitis includes mucosal colonization, microbial translocation of the mucous membrane, and invasion into the intravascular space, followed by intravascular survival and multiplication, reaching a high degree of bacteremia, translocation over the blood–brain barrier (BBB), and invasion of the meninges and central nervous system (CNS) [7,8]. The BBB provides a selective filter responsible for the exchange of water, iron, oxygen, nutrients, and other compounds between the CNS and the bloodstream [9]. The core element of the BBB is the cerebral blood vessel formed by endothelial cells [9]. Brain microvascular endothelial cells form many tight junctions (TJs) and adherent junctions with adjacent cells, resulting in proper paracellular permeability [10]. As the first line of defense restricting the paracellular passage of molecules, TJs are composed of different proteins, including integral membrane proteins (claudins, occludin, junctional adhesion molecules or “JAMs”) and peripheral membrane proteins (zonula occludins, such as ZO-1, ZO-2, and ZO-3) [11]. Studies have demonstrated that increased BBB leakage was associated with loss of TJ proteins and higher BBB-permeability biomarkers, including matrix metalloproteinases (MMP-9 and MMP-2), vascular endothelial growth factors (VEGFs), angiopoietin (ANG-1, ANG-2, ANG-3, and ANG-4), and endothelin-1 (ET-1), in brain injuries [12,13,14].

Quercetin, a flavonol compound found in plants, is a polyphenol and is widely distributed in fruits and vegetables [15]. Quercetin has been demonstrated to exhibit a broad spectrum of biological activities and properties such as anti-inflammatory, antibacterial, antiviral, and antioxidant [16,17,18]. As more studies have begun to focus on developing alternative drugs for problems associated with drug resistance and unfavorable side effects, more powerful functions of quercetin have been discovered [19]. Studies on reactive oxygen species (ROS)-induced carcinogenesis have reported that quercetin has anticancer activities, which were demonstrated to be related to specific molecular mechanisms, including MAPK/ERK, p53, JAK/STAT, TRAIL, AMPKα1/ASK1/p38, the RAGE/PI3K/AKT/mTOR axis, HMGB1, and NF-κB [20,21]. Quercetin may also protect vascular endothelium function via modulation of ROS/ADMA/DDAH II/eNOS/NO/Eph/Cav-1 signaling [22,23]. Due to its antioxidant and anti-inflammatory properties, quercetin also protects against cardiac and hepatic injuries in both acute and subacute rodent models [24,25]. Quercetin has also been reported to have anti-neuroinflammation and neuroprotective effects via the AMPK/NF-κB/NLRP3 inflammasome [26,27,28]. Although previous studies have revealed prominent properties of quercetin, whether quercetin can alleviate brain inflammation and protect BBB integrity during *G. parasuis* infection has not yet been studied.

Therefore, in this study, we established a mouse model of *G. parasuis* infection both in vivo and in vitro to unveil transcriptome changes in a mouse model of meningitis induced by *G. parasuis*. We investigated the protective effects of quercetin on brain inflammation by its ability to improve BBB integrity during *G. parasuis* infection.

## 2. Materials and Methods

### 2.1. Bacterial Strain and Drug

*G. parasuis* (GPS), SH0165, was provided by the State Key Laboratory of Agricultural Microbiology, Huazhong Agricultural University (Wuhan, China). The strain was isolated and cultivated in tryptic soy broth (Becton, Dickinson and Company, Franklin Lakes, NJ, USA) or tryptic soy agar (Becton, Dickinson and Company, Franklin Lakes, NJ, USA) with 10 µg/mL nicotinamide adenine dinucleotide (Beyotime Biotechnology, Wuhan, China) and 10% fetal bovine serum (Zhejiang Tianhang Biotechnology Co., Ltd., Huzhou, China).

Quercetin (CAS. 117-39-5, HPLC ≥ 98%) was obtained from Shanghai Yuanye Biotechnology Co., Ltd. (Shanghai, China). Quercetin was dissolved in DMSO and prepared in a 5 mg/mL quercetin storage solution and stored at –80 °C. Before use, the storage solution of quercetin was diluted 10 times with phosphate-buffered saline (PBS) to obtain a 500 µg/mL working solution, which was then diluted to a final concentration of 2.5 µg/mL, 5 µg/mL, 10 µg/mL, 20 µg/mL, and 40 µg/mL, based on a previous report [29].

### 2.2. Experimental Animals and Cells

Twenty healthy 6-week-old female Kunming mice were provided by Hubei Yi Zhicheng Biotechnology Co., Ltd. (Wuhan, China). Female mice were chosen according to our previous study of mice [30].

Brain microvascular endothelial cells (bEnd.3) were purchased from Warner Bio (Wuhan, China), cultured in complete Dulbecco’s Modified Eagle Medium (DMEM, Procell Life Science & Technology Co., Ltd., Wuhan, China) with 20% fetal bovine serum (Cell-box, Changsha, China), and cultivated in a humidified incubator of 95% air and 5% CO_2_ at 37 °C.

### 2.3. G. parasuis Infection and Quercetin Pretreatment

For the animal infection experiment, 20 6-week-old female Kunming mice were randomly assigned to two groups: the control group and the GPS infection group, with ten replicates per group. The GPS group was given 2 × 10^9^ CFU (CFU, colony forming unit) *G. parasuis* in 0.2 mL sterile saline by peritoneal injection, whereas the control group was injected with the equivalent amount of sterile saline [30]. The mice were euthanized and dissected after 24, 48, and 72 h of infection, and cerebrum tissues were isolated and kept at –80 °C for subsequent use.

To generate the cell infection model, bEnd.3 cells were seeded in 6-well plates (3 × 10^5^ cells/well). To confirm the optimal MOI of *G. parasuis*, bEnd.3 cells were infected by GPS with different MOIs (1:10, 1:1, 10:1, and 100:1) and different infection times (3 h, 6 h, and 12 h), with three replicates for each. The cells were then collected for RNA isolation, and the levels of inflammatory factors (*Il-1β*, *Il-6*, *Il-8*, *Il-18* and *Tnf-α*) were examined by qRT-PCR.

After constructing the cell infection model, an MOI of 1:1 and an infection time of 6 h were selected for the following quercetin pretreatment experiment. bEnd.3 cells were seeded in 6-well plates (3 × 10^5^ cells/well) and pretreated with quercetin at different concentrations (2.5 µg/mL, 5 µg/mL, and 10 µg/mL) for 2 and 3 h, respectively. After pretreatment with quercetin, bEnd.3 cells were infected with *G. parasuis* (MOI = 1:1) for 6 h, before isolating the cells for RNA and protein isolation.

### 2.4. RNA and Protein Extraction

Total RNA was extracted from mouse cerebrum tissue and bEnd.3 cells using TRIzol Universal total RNA extraction reagent, according to the manufacturer’s instructions (TIANGEN, Beijing, China). RNA was extracted by 1% agarose gel electrophoresis, and the concentration and purity of RNA were detected using a Merinton SMA5000 (Merinton, Beijing, China). Mouse cerebrum tissues and bEnd.3 cells were lysed with RIPA lysis buffer (G2038, Wuhan Servicebio Technology Co., Ltd., Wuhan, China) and placed on ice for 30 min. The lysate was centrifuged at 12,000 rpm for 15 min, and the supernatant was isolated and stored at −80 °C for future use. The total protein concentration was determined using a BCA protein detection kit (P0009, Beyotime Biotechnology, Shanghai, China).

### 2.5. Transcriptome Sequencing and Data Analysis

Total RNA was extracted using the RNeasy Mini Kit (Qiagen, Hilden, Germany) in accordance with the manufacturer’s instructions and the RIN number was checked to assess RNA integrity using an Agilent Bioanalyzer 2100 (Agilent Technologies, Inc., Santa Clara, CA, USA). Qualified total RNA was further purified using an RNAClean XP Kit (Beckman Coulter Inc., Fullerton, CA, USA) and RNase-Free DNase Set (Qiagen, Hilden, Germany). Only RNA with an RIN of ≥7.0 and a 28S/18S ratio of ≥0.7 was selected for deep sequencing. Libraries were generated using the VAHTS Total RNA-seq Library PrepKit for Illumina (Vazyme Biotech Co., Ltd., Nanjing, China) and subsequently sequenced using the Illumina HiSeq X-Ten platform.

The raw reads were filtered using the Seqtk sequence processing tool (https://github.com/lh3/seqtk (accessed on 5 eptember 2023)) before mapping to the reference genome (Ensembl_release110) using the Hisat2 alignment program. Gene fragments were counted using Stringtie (https://ccb.jhu.edu/software/stringtie/index.shtml (accessed on 8 September 2023)). Differentially expressed genes were identified using edgeR with a fold change of >2 and *p*-value of <0.05, which was considered as significantly differentially expressed.

GO and KEGG enrichment analyses were conducted on differentially expressed genes. GO terms with corrected *p*-values of ≤0.05 were considered significantly enriched. The KEGG automatic annotation server was used to perform pathway annotation of the entire genome as the background. Pathways with *p* ≤ 0.05 were considered to be significantly enriched.

Differentially expressed genes were analyzed using the STRING database (ELIXIR, Hinxton, Cambridgeshire, UK). A network of protein–protein interactions (PPIs) was built and visualized using Cytoscape software 3.9.1 (Institute for Systems Biology, Seattle, WA, USA). 

### 2.6. Cell Viability Assays

The bEnd.3 cells (1 × 10^4^ cells per well) were seeded into 96-well plates and treated with quercetin at final concentrations of 2.5 µg/mL, 5 µg/mL, 10 µg/mL, 20 µg/mL, and 40 µg/mL for 3, 6, 9, and 12 h. After treatment, bEnd.3 cells were incubated with the Cell Counting Kit-8 reagents (CCK-8, Beyotime Biotechnology, Shanghai, China) for 1 h, and the absorbance was measured at 450 nm using SpectraMax ABS plus (Molecular Devices, Shanghai, China).

### 2.7. Adhesion and Invasion Test of GPS

For the bacterial invasion test, bEnd.3 cells were seeded in 24-well plates (2 × 10^5^ cells/well) and infected with *G. parasuis* (MOI = 1:1) for 3, 6, and 9 h. The cells were then transferred to DMEM containing 10% double antibody and cultured for 1 h. Following culture, the cells were washed with PBS seven times and digested with 0.25% trypsin-EDTA solution at 37 °C for 5 min. The cells were collected and 1 mL of sterile ddH_2_O was added to lyse the cells. The lysate (200 μL) was cultured on the TSA plate, and the number of invasive bacteria was determined by gradient dilution counting for 48 h culture. The bacterial invasion index was calculated according to the following formula: intrusion index = the number of invasive bacteria/the number of bEnd.3 cells. 

For the bacterial adhesion test, the cells were cultured in DMEM without antibody after GPS infection. Subsequently, the cells went through the same processing procedure as the previous invasion test. The bacterial number determined by gradient dilution includes adhesive and invasive bacteria. The bacterial adhesion index was calculated according to the following formula: intrusion index = (number of adhesive and invasive bacteria − number of invasive bacteria)/number of bEnd.3 cells.

### 2.8. Hematoxylin and Eosin (H&E) Staining and Immunofluorescence Microscopy

Mouse brain histopathology was evaluated via H&E staining. The mouse brain was fixed in 4% paraformaldehyde and embedded in paraffin. The sections (4 µm) were stained with H&E using the standard method and observed under a microscope.

The bEnd.3 cells (2 × 10^5^ cells per well) were placed on a 6-well plate. After treatment with quercetin and infection with GPS, the cells were soaked in 4% paraformaldehyde for 10 min, blocked with 5% bovine serum albumin (BSA) at room temperature for 1 h, and incubated with the following primary antibodies: ZO-1 (A0659, 1:100, ABclonal, Wuhan, China), Claudin-5 (A10207, 1:100, ABclonal, Wuhan, China), and Occludin (27260-1-AP, 1:800, Wuhan Sanying Biotechnology Co., Ltd., Wuhan, China), at 4 °C overnight. The cells were then incubated with FITC goat anti-rabbit IgG (H+L) (AS011, 1:100, ABclonal, Wuhan, China) at room temperature for 1 h, stained with DAPI anti-fading adherent medium, and observed in an EVOS FL Auto system (Thermo Fisher Scientific, Shanghai, China).

### 2.9. The qRT-PCR Analysis

The cDNA was synthesized according to the instructions of the Primescript^®^ RT Reagent Kit with gDNA Eraser (TaKaRa, Beijing, China). qRT–PCR was performed using the TB Green^®^ Premix Ex TaqTM kit (TaKaRa, Beijing, China). Primers were designed using Primer Premier 5.0 software (Table 1). At least three technical repeats were amplified for each sample, with GAPDH as the internal reference gene. The relative-expression fold change was calculated using the 2^−ΔΔCt^ method. Primer efficiency was calculated and only primers with efficiency 95~110% were utilized for qRT-PCR. 

### 2.10. Western Blotting

Proteins were separated by 12% SDS-PAGE and transferred to a PVDF membrane. Following blocking with 5% skim milk, the blots were incubated with primary antibodies or GAPDH for 6 h. After washing with TBST five times, the blots were incubated with HRP Goat Anti-Mouse IgG (H+L) (ABclonal, Wuhan, China) for 30 min and subsequently treated with the reagents from the ECL Enhanced Kit (ABclonal, Wuhan, China). The protein expression levels of Sema4D, PlexinB1, PI3K, p-PI3K, Akt, p-Akt, Erk, p-Erk, and Gapdh were quantified using the FluorChem™ FC2 AIC system (Alpha Innotech, San Leandro, CA, USA).

### 2.11. Statistical Analysis

The experimental data are expressed as the mean ± SD. We performed the Shapiro–Wilk normality test on experimental data. For data with no significant deviation from normal distribution, the two-tailed Student’s *t* test was utilized for difference analysis. And a non-parametric Mann–Whitney rank sum test was used for data that did not follow normal distribution. Differences with *p* < 0.05 indicate statistical significance.

## 3. Results

### 3.1. GPS-Induced Meningitis Traits in Mice

**Figure 1:**
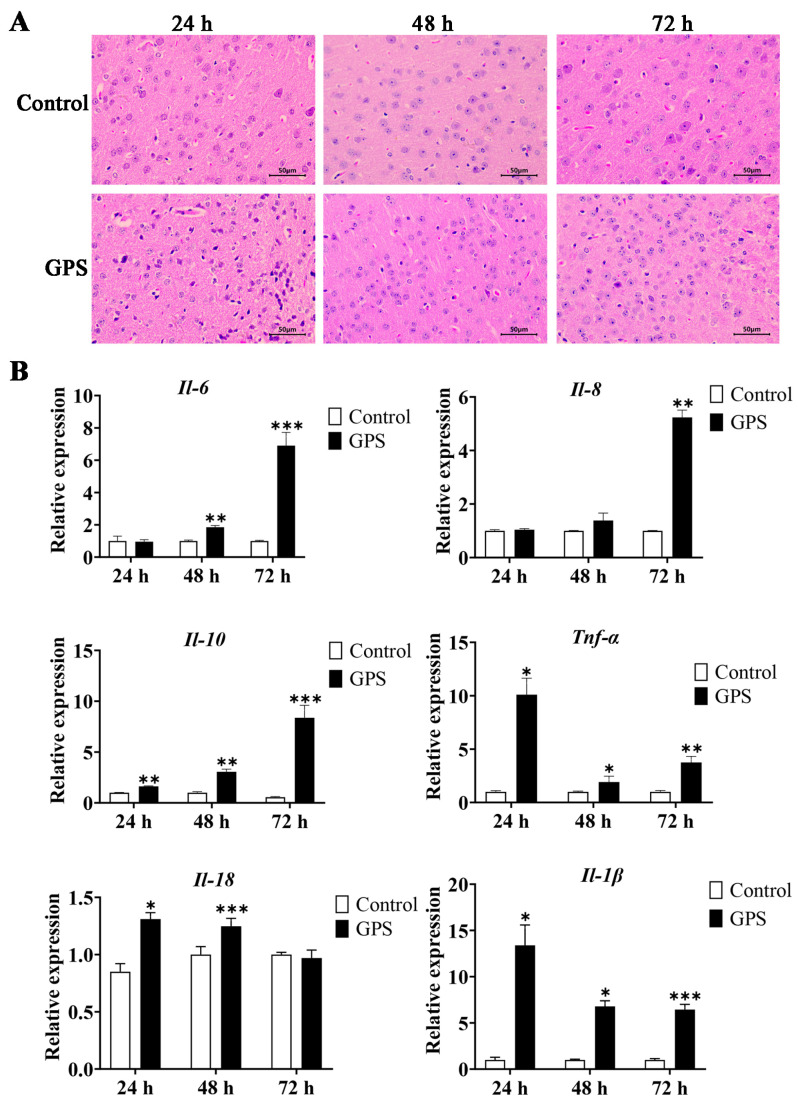
Cerebral inflammation and meningeal shedding in *G. parasuis*-infected mice. (**A**) H&E staining of mouse cerebral tissue after 24, 48, and 72 h infection with GPS (magnification 10 × 40). (**B**) Expression levels of inflammatory factors in the mouse cerebrum after GPS infection. Data are presented as the mean ± SD (n = 3). GPS: *G. parasuis*. * *p* < 0.05, ** *p* < 0.01, and *** *p* < 0.001, versus the control group.

After 24, 48, and 72 h infection with GPS, the mouse was dissected and the brain tissue samples were collected for histopathological section analysis. The results showed that the meninges of the infected mice exhibited typical meningitis symptoms with obvious shedding (Supplementary Figure S1). The types and quantities of inflammatory cells increased, showing obvious infiltration of inflammatory cells in the GPS-infected mice cerebrum (Figure 1A). In addition, the mRNA expression levels of inflammatory cytokines (*Il-6*, *Il-8*, *Il-10*, *Il-18*, *Il-1β*, and *Tnf-α*) in GPS-infected mice were significantly increased compared to the control group (*p* < 0.05, Figure 1B), which indicated that GPS successfully induced brain inflammation in mice.

### 3.2. Transcriptome Changes and Significant Pathways in the Cerebrum of GPS-Infected Mice

**Figure 2:**
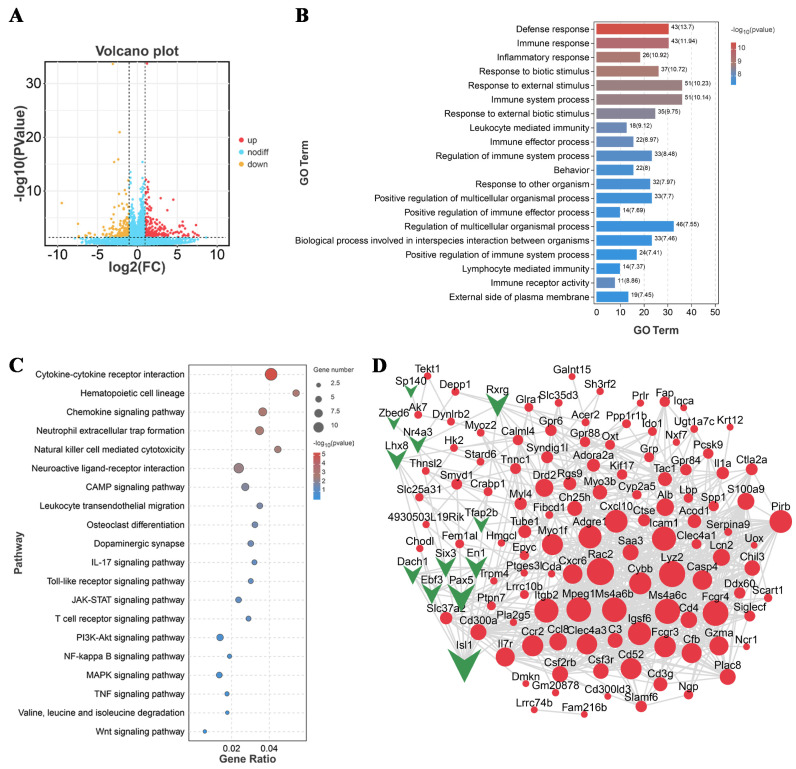
Deep sequencing revealed the transcriptional profiles in the cerebrum of GPS-infected mice. (**A**) Volcano plot showing DEGs in the cerebrum of GPS-infected mice. Red dots represent upregulated genes and yellow dots represent downregulated genes. (**B**) GO-enrichment analysis for DEGs. (**C**) KEGG pathways significantly enriched by DEGs in the GPS infection group. (**D**) PPI network constructed by DEGs in the GPS infection group. Upregulated expression is indicated by red, and downregulated expression is indicated by green. The node degree evaluating the interaction frequency is represented by node size. GPS: *G. parasuis*.

Transcriptome sequencing was performed on the brain tissue from the GPS infection group (72 h) and control group. Differential expression analysis showed that 161 genes were significantly differentially expressed in the GPS infection group (*p* < 0.05) compared to the control group. Among these differentially expressed genes (DEGs), 86 genes were significantly upregulated and 75 genes were significantly downregulated after GPS infection (*p* < 0.05, Figure 2A). All 161 DEGs were subsequently used for GO- and KEGG-enrichment analyses. The results of GO analysis revealed that the DEGs induced by GPS were mainly involved in the defense response, immune response, inflammatory response, response to external stimulus, response to biotic stimulus, and the immune system process (Figure 2B). KEGG enrichment showed that cytokine–cytokine receptor interaction, neuroactive ligand interaction, PI3K-Akt signaling pathway, MAPK signaling pathway, JAK-STAT signaling pathway, and NF-kappa B signaling pathway were the major affected pathways (Figure 2C). To reveal the interactions between the DEGs in the GPS infection group, a protein–protein interaction (PPI) network was constructed by STRING analysis. The PPI network consisted of 124 differential proteins (nodes) and 760 interactions (edges) between these differential proteins (Figure 2D). The node degree was used to evaluate the crucial roles of proteins, and the top 20 proteins with the highest connection degrees were highlighted within the network, including Rac2, Lyz2, Fcgr4, Ms4a6c, Ms4a6b, Mpeg1, Itgb2, Icam1, Adgre1, Igsf6, Cxcl10, Pirb, Cybb, Fcgr3, Cd52, Myolf, Ccr2, Clec4a3, Cfb, and Casp4.

### 3.3. GPS Infection Decreased the Expression of Angiogenetic Genes

**Figure 3:**
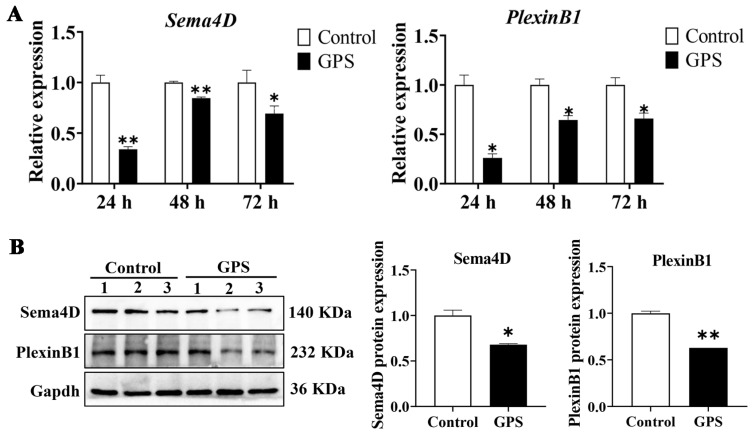
Validation of the differential expression of angiogenetic genes (*Sema4D* and *PlexinB1*) in the cerebrum of GPS-infected mice (24, 48, and 72 h). (**A**) qRT–PCR validation for mRNA expression changes of *Sema4D* and *PlexinB1*. (**B**) Western blot validation for protein expression changes of Sema4D and PlexinB1 after 72 h of GPS infection, original western blots are available in the Supplementary Materials. Data are presented as the mean ± SD (n = 3). GPS: *G. parasuis*. * *p* < 0.05, and ** *p* < 0.01, versus the control group.

After transcriptome analysis, we validated the expression of the angiogenetic genes *Sema4D* and *PlexinB1* in the cerebrum of mice infected with GPS for 24, 48, and 72 h. The qRT-PCR showed that the mRNAs of *Sema4D* and *PlexinB1* in the GPS-infected group were significantly downregulated compared to those in the control group (*p* < 0.05, Figure 3A). The protein expression of Sema4D and PlexinB1 was subsequently validated by Western blotting. The results showed that Sema4D and PlexinB1 were significantly downregulated after 72 h of GPS infection (*p* < 0.05, Figure 3B). These results suggested that GPS infection attenuated angiogenesis in the mouse cerebrum.

### 3.4. GPS Infection Disrupted the TJ of the Mouse Cerebrum In Vivo

**Figure 4:**
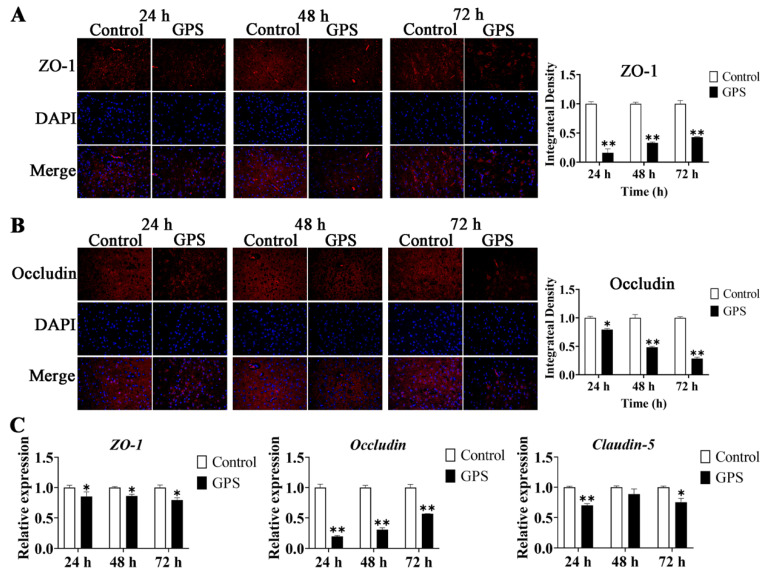
GPS infection disrupted the tight junction of the mouse cerebrum. Immunofluorescence staining and integrated density of ZO-1 (**A**) and Occludin (**B**) in the mouse cerebrum (magnification 10 × 40). (**C**) Relative expression of *ZO-1*, *Occludin*, and *Claudin-5* in the mouse cerebrum (mean ± SD, n = 3). GPS: *G. parasuis*. * *p* < 0.05 and ** *p* < 0.01, versus control group.

To confirm the effects of GPS infection on the BBB integrity of the mouse cerebrum, the organization and distribution images of ZO-1 and occludin in the mouse cerebrum were detected using immunofluorescence (IF). GPS infection significantly altered the distribution patterns of ZO-1 and Occludin (Figure 4A,B). The integrated density of fluorescence was quantified using densitometric analysis in ImageJ 1.8.0. Staining for ZO-1 and Occludin in the cerebrum of GPS-infected mice (24, 48, and 72 h) appeared to be significantly reduced and fragmented compared to that of the control group (*p* < 0.01; *p* < 0.05) (Figure 4A,B). The mRNA expression of *ZO-1*, *Occludin*, and *Claudin-5* was determined by qRT–PCR in the cerebrum of GPS-infected mice (24, 48, and 72 h). The results showed that GPS infection significantly decreased the expression of *ZO-1* (*p* < 0.05), *Occludin* (*p* < 0.01), and *Claudin-5* (*p* < 0.05) in the mouse cerebrum (Figure 4C). These results indicated that GPS infection could disrupt the TJs in the mouse cerebrum in vivo.

### 3.5. GPS Infection Promoted the Expression of BBB-Permeability Marker Genes

**Figure 5:**
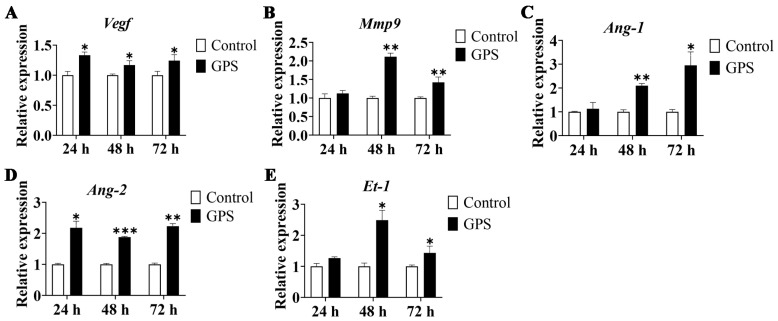
Relative expression of BBB-permeability marker genes in the cerebrum of GPS-infected mice (mean ± SD, n = 3). (**A**) Vegf, (**B**) Mmp9, (**C**) Ang-1, (**D**) Ang-2, and (**E**) Et-1. GPS: *G. parasuis*. * *p* < 0.05, ** *p* < 0.01, and *** *p* < 0.001, versus the control group.

To assess whether GPS infection could destroy the BBB of the mouse cerebrum, the mRNA expression levels of the BBB-permeability marker genes (*Vegf*, *Mmp9*, *Ang-1*, *Ang-2*, and *Et-1*) were determined by qRT-PCR. The results showed that *Vegf*, *Mmp9*, *Ang-1*, *Ang-2*, and *Et-1* were significantly upregulated after GPS infection for 48 and 72 h (*p* < 0.05). Vegf and *Ang-2* were also significantly increased after GPS infection for 24 h (*p* < 0.05) (Figure 5). These results suggested that GPS infection increased the BBB permeability of the mouse cerebrum.

### 3.6. GPS Infection Suppressed the Activation of PI3K/Akt/Erk Pathways In Vivo

**Figure 6:**
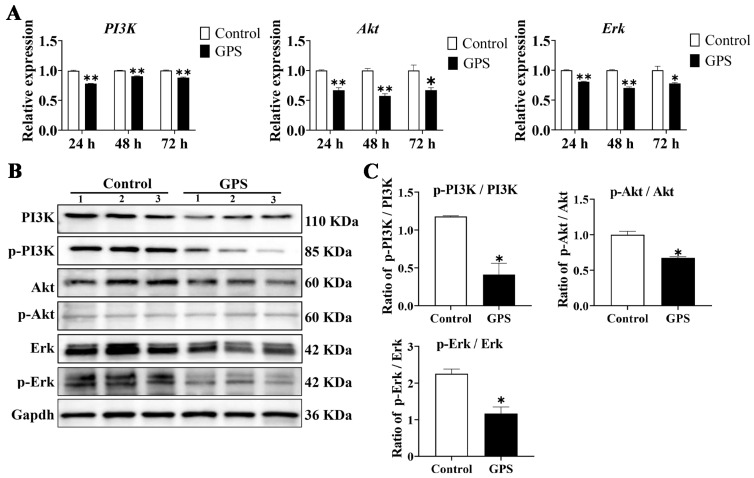
Effect of GPS infection on the activation of PI3K/Akt/Erk pathways in vivo. (**A**) Relative mRNA expression of *PI3K*, *Akt*, and *Erk* in the cerebrum of GPS-infected mice (mean ± SD, n = 3). (**B**) Western blot results of PI3K, p-PI3K, Akt, p-Akt, Erk, and p-Erk in the cerebrum of mice after 72 h of GPS infection, original western blots are available in the Supplementary Materials. (**C**) Ratio of phosphorylated protein to non-phosphorylated protein (PI3K, Akt, and Erk) in the GPS-infected group. GPS: *G. parasuis*. * *p* < 0.05 and ** *p* < 0.01, versus control group.

The expression and activation of the PI3K/Akt/Erk pathways were detected by qRT-PCR and Western blotting, respectively. As shown in Figure 6A, the mRNA expression of *PI3K*, *Akt*, and *Erk* in the cerebrum of GPS-infected mice (24, 48, and 72 h) was significantly decreased compared to that in the control group (*p* < 0.05). The protein expression levels of PI3K, p-PI3K, Akt, p-Akt, Erk, and p-Erk in the mouse cerebrum were detected by Western blotting (Figure 6B). The results showed that, after 72 h of GPS infection, the phosphorylation of PI3K (p-PI3K/PI3K) in the GPS-infected group was significantly decreased (*p* < 0.01), as was the phosphorylation of Akt and Erk (p-Akt/Akt and p-Erk/Erk) (*p* < 0.05) compared to the control group (Figure 6C).

### 3.7. GPS Infection Model in Mouse Brain Microvascular Endothelial Cells (bEnd.3)

**Figure 7:**
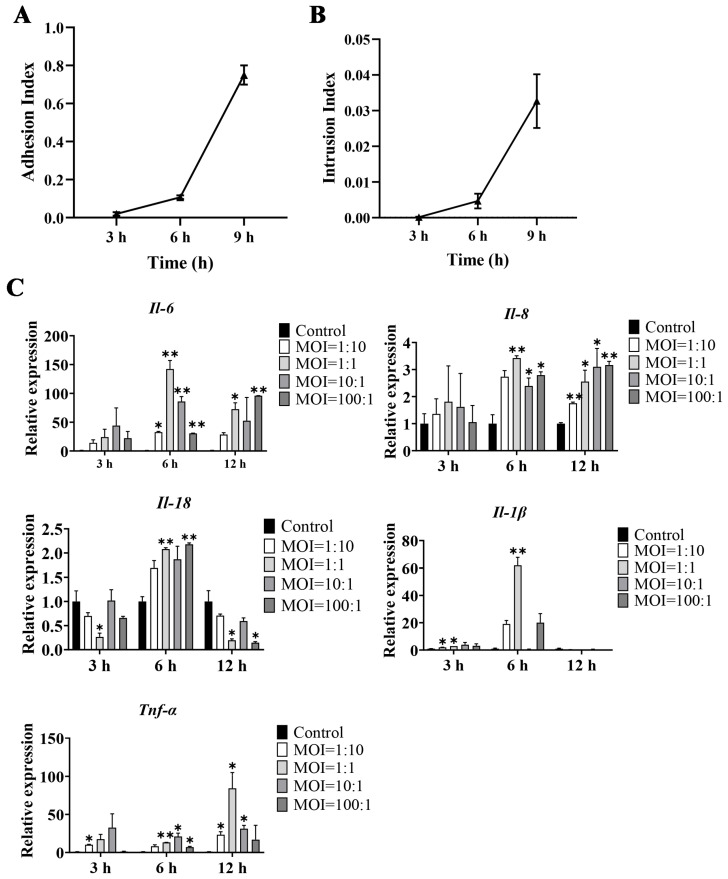
GPS infection model in mouse brain microvascular endothelial cells. Adhesion (**A**) and intrusion (**B**) of GPS to bEnd.3 cells (mean ± SD, n = 3). (**C**) Changes in the expression of inflammatory factors (*Il-6*, *Il-8*, *Il-18*, *Il-1β*, and *Tnf-α*) in GPS-infected bEnd.3 cells (mean ± SD, n = 3). The horizontal axis represents the infection time of GPS. MOI: Multiplicity of infection; GPS: *G. parasuis*; * *p* < 0.05 and ** *p* < 0.01, versus control group.

We next constructed an in vitro GPS infection model in mouse brain microvascular endothelial cells (bEnd.3), and determined the adhesion and invasion indices. When bEnd.3 cells were infected with GPS for 3 h (MOI = 1:1), the average adhesion index was 1.93% and the invasion index was approximately 0.02%; when bEnd.3 cells were infected with GPS for 6 h (MOI = 1:1), the average adhesion index was 10.73% and the invasion index was 0.47%; and when bEnd.3 cells were infected with GPS for 9 h (MOI = 1:1), the average adhesion index increased to 75% and the invasion index increased to 3.27% (Figure 7A,B).

The bEnd.3 cells were infected by GPS with different MOIs (1:10, 1:1, 10:1, and 100:1) and infection times (3 h, 6 h, and 12 h). Subsequently, the relative expression of inflammatory factors (*Il-6*, *Il-8*, *Il-18*, *Il-1β*, and *Tnf-α*) was detected by qRT–PCR. The results showed that all of these inflammatory cytokines were upregulated after GPS infection (Figure 7C). In particular, *Il-6* was significantly upregulated after 6 h of infection (MOI = 1:10, 1:1, 10:1, and 100:1) and 12 h infection (MOI = 1:1 and 100:1) (*p* < 0.05, Figure 7C). *Il-8* was significantly upregulated after 6 h infection (MOI = 1:1, 10:1, and 100:1) and 12 h infection (MOI = 1:10, 1:1, 10:1, and 100:1) (*p* < 0.05, Figure 7C). Similarly, *Il-18* and *Tnf-α* showed significant upregulation after 3, 6, and 12 h of infection (*p* < 0.05, Figure 7C), and *Il-1β* showed significant upregulation after 3 h (MOI = 1:10 and 1:1) and 6 h (MOI = 1:1) of infection (*p* < 0.05, Figure 7C). Thus, an MOI of 1:1 and an infection time of 6 h were used to construct the GPS infection model. 

### 3.8. Structure and Cytotoxicity of Quercetin

**Figure 8:**
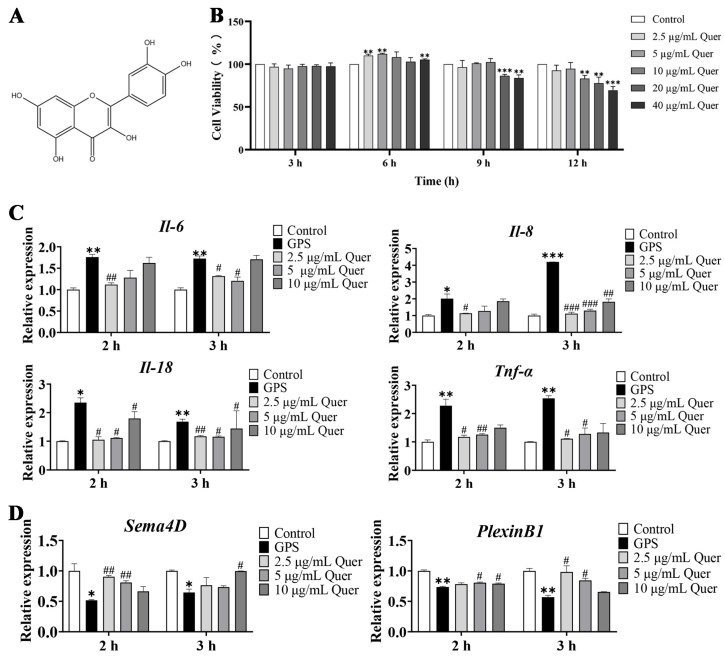
Effects of quercetin on the cytotoxicity, inflammation, and angiogenesis of bEnd.3 cells. (**A**) Structure of quercetin. (**B**) Cytotoxicity of quercetin on bEnd.3 cells at different concentrations (2.5 µg/mL, 5 µg/mL, 10 µg/mL, 20 µg/mL, and 40 µg/mL). (**C**) Effects of quercetin (2.5 µg/mL, 5 µg/mL, and 10 µg/mL) on inflammatory factors (*Il-6*, *Il-8*, *Il-18*, and *Tnf-α*) in GPS-infected bEnd.3 cells (mean ± SD, n = 3). (**D**) Effects of quercetin (2.5 µg/mL, 5 µg/mL, and 10 µg/mL) on angiogenetic genes (*Sema4D* and *PlexinB1*) in GPS-infected bEnd.3 cells (mean ± SD, n = 3). The horizontal axis represents the pretreating time of quercetin. Quer: Quercetin, GPS infection: MOI = 1:1, 6 h; GPS: *G. parasuis*; * *p* < 0.05, ** *p* < 0.01, and *** *p* < 0.001, versus the control group; ^#^ *p* < 0.05, ^##^ *p* < 0.01, and ^###^ *p* < 0.001, versus the GPS group.

Quercetin (C_15_H_10_O_7_) is a widely distributed flavonol compound with a variety of biological activities in the plant kingdom (Figure 8A). The bEnd.3 cells were treated with different concentrations of quercetin (2.5 µg/mL, 5 µg/mL, 10 µg/mL, 20 µg/mL and 40 µg/mL) for 3, 6, 9, and 12 h. The test for cell viability showed that 3 h incubation with quercetin had no significant cytotoxicity on bEnd.3 cells (Figure 8B). A 6 h incubation with quercetin improved bEnd.3 cell viability at concentrations of 2.5 µg/mL, 5 µg/mL, and 40 µg/mL (*p* < 0.01, Figure 8B). If the incubation time was extended to 9 h and 12 h, cytotoxicity was observed at higher concentrations of quercetin (20 µg/mL and 40 µg/mL for 9 h; 10 µg/mL, 20 µg/mL, and 40 µg/mL for 12 h) (*p* < 0.01, Figure 8B). 

### 3.9. Quercetin Suppressed GPS-Induced Inflammatory Responses in bEnd.3 Cells

Based on the previous cell viability results, concentrations of 2.5 µg/mL, 5 µg/mL, and 10 µg/mL and incubation times of 2 and 3 h were used in the following quercetin pretreatment experiments. After pretreatment with quercetin, bEnd.3 cells were infected with GPS (MOI = 1:1) for 6 h. The qRT-PCR results showed that the relative expression of *Il-6*, *Il-8*, *Il-18*, and *Tnf-α* in the GPS-infected group was significantly upregulated (*p* < 0.05, Figure 8C). Furthermore, quercetin significantly decreased the expression levels of *Il-6*, *Il-8*, *Il-18* and *Tnf-α*, compared to the GPS infection group (*p* < 0.05, Figure 8C). 

### 3.10. Quercetin Increased the Expression of Angiogenetic Genes in bEnd.3 Cells

We also detected the mRNA expression of angiogenetic genes (*Sema4D* and *PlexinB1*) in bEnd.3 cells. The results showed that GPS infection significantly decreased the expression levels of *Sema4D* and *PlexinB1* (*p* < 0.05, Figure 8D), whereas quercetin increased the expression levels of these angiogenetic genes to a normal level (Figure 8D). 

### 3.11. GPS-Induced TJ Disruption In Vitro

**Figure 9:**
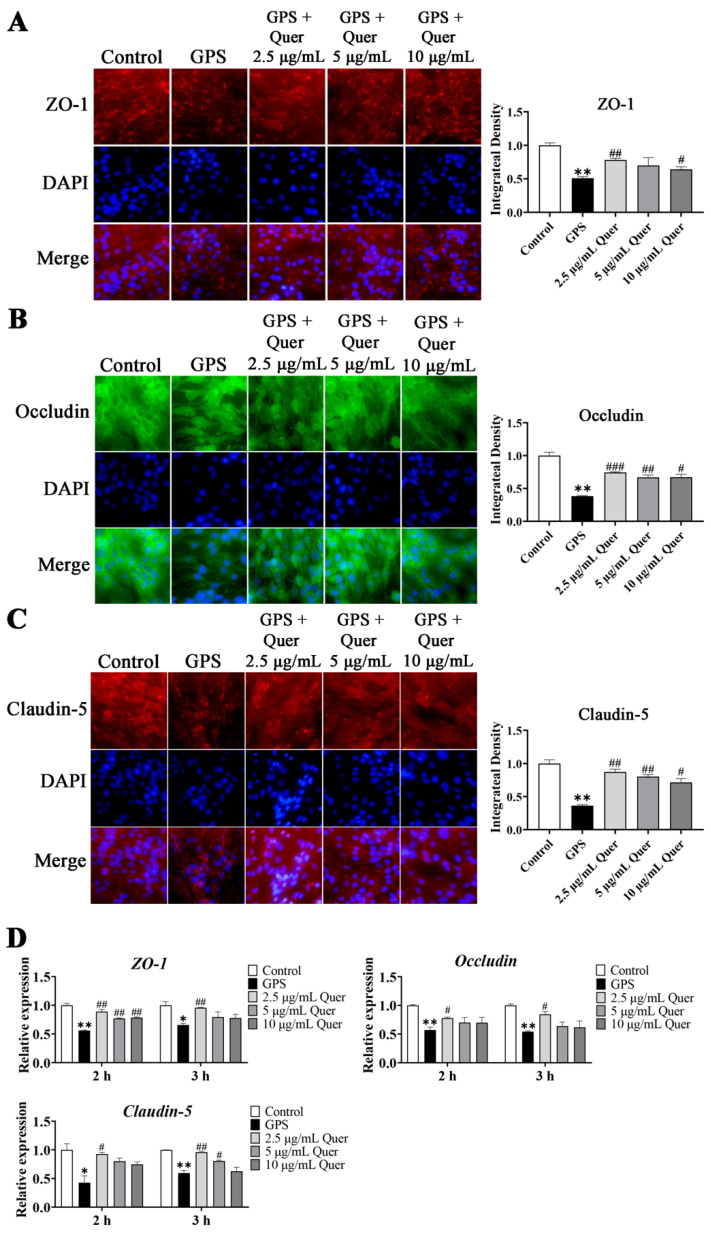
Quercetin improved GPS-induced downregulation of tight junctions in bEnd.3 cells. (**A**) Immunofluorescence of ZO-1 (red signal). (**B**) Immunofluorescence of Occludin (green signal). (**C**) Immunofluorescence of Claudin-5 (red signal). Immunofluorescence images were obtained for bEnd.3 cells pretreated with quercetin for 3 h. (**D**) Quantitative mRNA expression of *ZO-1*, *Occludin*, and *Claudin-5* (mean ± SD, n = 3). Quer: Quercetin; 2 and 3 h: time of quercetin pretreatment; GPS infection: MOI = 1:1, 6 h; GPS: *G. parasuis*; * *p* < 0.05 and ** *p* < 0.01, versus control group; ^#^ *p* < 0.05, ^##^ *p* < 0.01, and ^###^ *p* < 0.001, versus infection group.

To confirm the effects of quercetin on protecting bEnd.3 cell monolayers against GPS-induced degradation of TJ proteins, we used immunofluorescence assays to determine the intercellular junctions of ZO-1, Occludin, and Claudin-5 proteins in bEnd.3 cells. Immunofluorescence intensity showed that the intercellular junctions of ZO-1, Occludin, and Claudin-5 were disrupted by GPS (MOI = 1:1, 6 h) (*p* < 0.01, Figure 9A–C). Quercetin improved the integrity of ZO-1 at 2.5 µg/mL, significantly at 10 µg/mL (*p* < 0.05, Figure 9A). Quercetin also significantly improved the integrity of Occludin and Claudin-5 at 2.5 µg/mL, 5 µg/mL, and 10 µg/mL (*p* < 0.05, Figure 9B,C). The mRNA expression of the TJ proteins in bEnd.3 cells showed the same regulatory pattern, in that GPS decreased the expression of *ZO-1*, *Occludin*, and *Claudin-5* (*p* < 0.01, Figure 9D), and quercetin increased the expression of these TJs (*p* < 0.05, Figure 9D), with 2.5 µg/mL and a 2 h treatment of quercetin exhibiting the best effect. The results indicated that quercetin had a protective effect on TJs in bEnd.3 cells.

### 3.12. Quercetin Suppressed GPS-Induced Upregulation of BBB-Permeability Marker Genes

**Figure 10:**
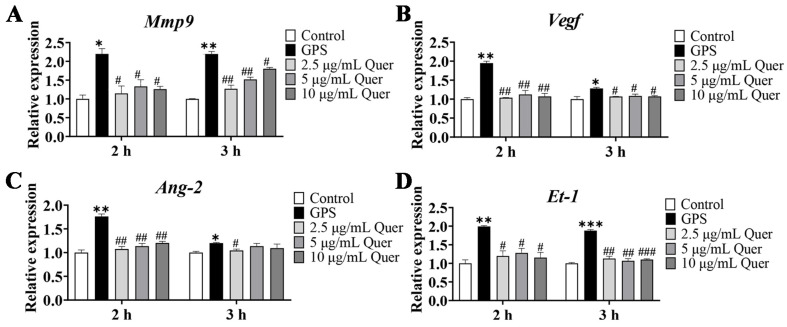
Quercetin decreased the expression of BBB-permeability marker genes in GPS-infected bEnd.3 cells (mean ± SD, n = 3). (**A**) *Mmp9*, (**B**) *Vegf*, (**C**) *Ang-2*, and (**D**) *Et-1*. Quer: Quercetin; 2 and 3 h: time of quercetin pretreatment; GPS infection: MOI = 1:1, 6 h; GPS: *G. parasuis*; * *p* < 0.05, ** *p* < 0.01, and *** *p* < 0.001, versus control group; ^#^ *p* < 0.05, ^##^ *p* < 0.01, and ^###^ *p* < 0.001, versus infection group.

To investigate whether quercetin ameliorated the BBB permeability induced by GPS, the expression levels of BBB-permeability marker genes Mmp9, *Vegf*, *Ang-2*, and *Et-1* were detected in bEnd.3 cells. Consistent with the results in cerebrum tissues, the expression of *Mmp9*, *Vegf*, *Ang-2*, and *Et-1* genes in GPS-infected bEnd.3 cells were significantly increased (*p* < 0.05), whereas quercetin decreased the upregulation of *Mmp9*, *Vegf*, *Ang-2*, and *Et-1* induced by GPS infection in bEnd.3 cells (*p* < 0.05, Figure 10A–D), indicating that quercetin could improve the BBB permeability induced by GPS.

### 3.13. Quercetin Reactivated GPS-Induced Suppression of the PI3K/Akt/Erk Pathway In Vitro

**Figure 11:**
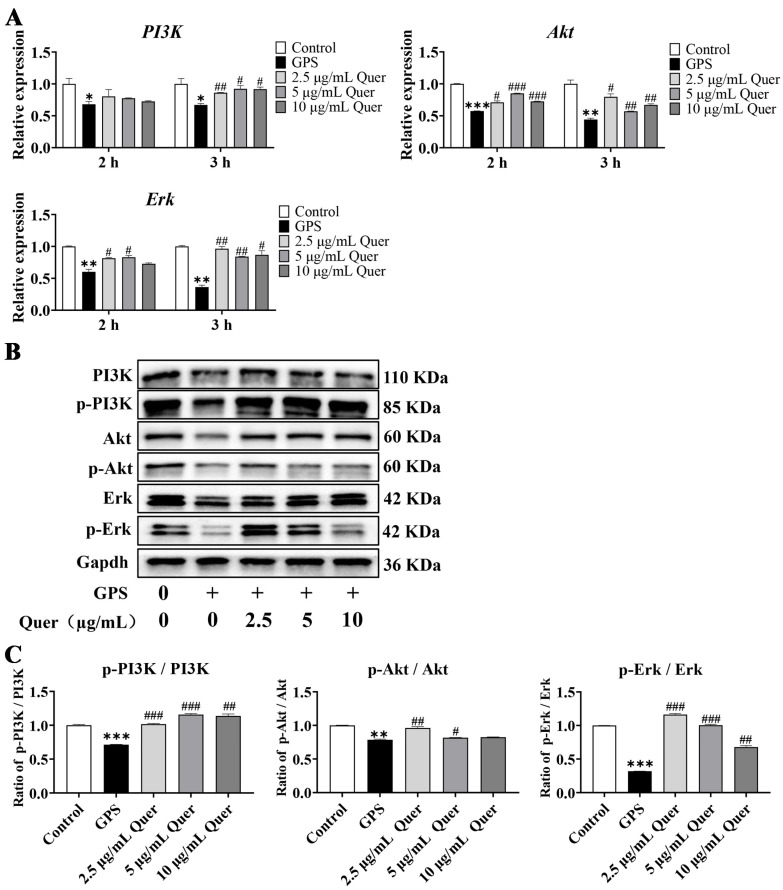
Quercetin reactivated the PI3K/Akt/Erk pathway in GPS-infected bEnd.3 cells. (**A**) mRNA expression of *PI3K*, *Akt*, and *Erk* genes (mean ± SD, n = 3). The horizontal axis (2 h and 3 h) represents the pretreatment time of quercetin. (**B**) The protein expression of PI3K, p-PI3K, Akt, p-Akt, Erk, and p-Erk, original western blots are available in the Supplementary Materials. (**C**) The phosphorylation ratio of PI3K, Akt, and Erk detected by the grayscale value of protein bands. GPS infection: MOI = 1:1, 6 h; Quer: Quercetin; GPS: *G. parasuis*; * *p* < 0.05, ** *p* < 0.01, and *** *p* < 0.001, versus control group; ^#^ *p* < 0.05, ^##^ *p* < 0.01, and ^###^ *p* < 0.001, versus infection group.

To further explore the role of the PI3K/Akt/Erk pathway in the effects of quercetin, the mRNA and protein expression of PI3K/Akt/Erk were detected by qRT-PCR and Western blotting. Compared to the control group, *PI3K*, *Akt*, *and Erk* mRNA levels were significantly decreased in GPS-infected bEnd.3 cells (*p* < 0.05, Figure 11A). Furthermore, a 3 h treatment with quercetin significantly upregulated the mRNA expression of *PI3K*, *Akt*, and *Erk* (*p* < 0.05, Figure 11A), but a 2 h treatment with quercetin showed no significant effect on the mRNA expression of *PI3K*. Thus, bEnd.3 cells pretreated with quercetin for 3 h were subsequently used for Western blotting. The results showed that the protein expression of PI3K, p-PI3K, Akt, p-Akt, Erk, and p-Erk decreased in the GPS infection group and was upregulated in the quercetin groups, with 2.5 µg/mL quercetin showing the best effect (Figure 11B). The grayscale values of protein bands showed that the phosphorylation ratios of PI3K, Akt, and Erk were significantly decreased in the GPS infection group and significantly increased in the quercetin groups, with 2.5 µg/mL quercetin exhibiting the best effect (*p* < 0.001 for 2.5 µg/mL quercetin, Figure 11C). The results suggested that quercetin reactivated GPS-induced suppression of the PI3K/Akt/Erk pathway in vitro.

## 4. Discussion

In this study, we established a mouse model of *G. parasuis* infection in vivo and *in vitro*, with the aim of revealing the transcriptome changes induced during *G. parasuis* infection and investigating the protective effects of quercetin on BBB integrity. We demonstrated that *G. parasuis* infection caused brain inflammation in mice, destroyed TJs, and suppressed the activation of the PI3K/Akt/Erk signaling pathway. In the *G. parasuis*-infected bEnd.3 cells, we found that quercetin could reduce the levels of inflammatory cytokines (*Il-18*, *Il-6*, *Il-8*, and *Tnf-α*) and BBB-permeability marker genes (*Mmp9*, *Vegf*, *Ang-2* and *Et-1*), promote the expression of angiogenetic genes (*Sema4D* and *PlexinB1*), alleviate the disruption of the integrity of the bEnd.3 cell monolayers, upregulate the expression of *ZO-1*, *Occludin*, and *Claudin-5*, and promote the activation of the PI3K/Akt/Erk pathway.

Inflammatory cytokines serve as crucial mediators in the pathogenesis of inflammation, immunity, and disease [31]. Studies have demonstrated that in piglets infected with *G. parasuis*, mononuclear phagocytes increase the gene expression levels of the inflammatory cytokines *IL-6*, *IL-8*, and *IL-10* via the NF-κB signaling pathway [32]. *G. parasuis* infection also triggers the upregulation of inflammatory cytokines, IL-1α, IL-1β, IL-6, IL-8, and TNF-α, in porcine alveolar macrophages by activating NF-κB and MAPK signaling pathways [33,34]. Moreover, *G. parasuis* elevated the expression of pro-inflammatory cytokines IL-1β, IL-6, and TNF-α in mice through the P38 and JNK-MAPK signaling pathways [35]. Therefore, these inflammatory cytokines play a pivotal role in the inflammatory responses following *G. parasuis* infection. Quercetin, a flavonoid compound, exhibits antibacterial, anti-inflammatory, and various other biological activities, making it highly valuable in the field of medicine [26]. Quercetin treatment has been reported to reduce oxidative stress and inflammation by downregulating the expression of inflammatory cytokines (TNF-α, IL-1, and IL-6) in LPS-induced lung epithelial cells [36]. Quercetin has also been reported to mitigate *Pseudomonas aeruginosa*-induced acute lung inflammation by decreasing the production of proinflammatory cytokines such as IL-1β, IL-6, and TNF-α [37]. Furthermore, quercetin treatment has been shown to notably lower the mRNA expression levels of proinflammatory cytokines (TNF-α, IL-1β, and IL-6) in lipopolysaccharide (LPS)-induced bovine intestinal epithelial cells (BIECs) [38]. We examined the changes in mRNA expression of inflammatory factors (*Il-1β*, *Il-6*, *Il-8*, *Il-10*, *Il-18*, and *Tnf-α*) in *G. parasuis*-infected mice, both in vivo and in vitro. The findings confirmed that *G. parasuis* infection significantly increased the expression of *Il-1β*, *Il-6*, *Il-8*, *Il-10*, *Il-18*, and *Tnf-α* in mice and mouse brain microvascular endothelial cells (bEnd.3). Moreover, quercetin effectively suppressed the upregulation of *Il-8*, *Il-6*, *Il-18*, and *Tnf-α* in *G. parasuis*-infected bEnd.3 cells, thus mitigating the occurrence of brain inflammation.

PI3K/Akt/Erk is an important signaling pathway involved in inflammatory responses during ischemic brain injury [39]. It has been reported that, after infection with *G. parasuis*, the expression of p38, ERK, and JNK increased in porcine alveolar macrophages (PAMs) via the NF-κB and MAPK signaling pathways, leading to the upregulation of inflammatory cytokines such as IL-1α, IL-1β, IL-6, IL-8, and TNF-α [33]. *G. parasuis* infection can also induce PAMs to produce IL-17 by activating the PKC-ERK/MAPK and IκB/NF-κB signaling pathways [40]. Our previous study confirmed that *G. parasuis* induces immunosuppression and upregulation of inflammatory cytokines via the PI3K/Akt/mTOR signaling pathway in piglets [41]. Conversely, quercetin has been reported to modulate inflammatory cytokines and improve neurological defects by targeting the PI3K/Akt/NF-κB and MEK/ERK pathways in LPS-challenged mice [42]. Furthermore, quercetin has shown promise in treating vascular disease by suppressing inflammatory responses and apoptosis through the PI3K/Akt signaling pathway [43]. It has also been reported that quercetin could attenuate arthritis by targeting the HMGB1/TLR4/p38/ERK1/2/NF-κB p65 pathway in mice [44]. Overall, we concluded from these reports that the PI3K/Akt/Erk signaling pathway plays an important role in *G. parasuis*-induced brain inflammation and that quercetin has a potential therapeutic effect on mitigating inflammatory responses via the PI3K/Akt/Erk signaling pathway.

TJs are composed of cytoplasmic proteins, transmembrane proteins, and connective adhesion molecules between capillary endothelial cells, and are important in maintaining the BBB integrity [45]. ZO-1, Occludin, and Claudin-5 are the most important proteins in TJs. Increased BBB permeability is usually associated with changes in ZO-1, Occludin, and Claudin-5 [46].The expression of TJ proteins (ZO-1, Occludin, and Claudin-5) is significantly reduced in LPS-stimulated human microvascular endothelial cells (HBMECs), resulting in BBB dysfunction [47]. Previous studies have reported that *G. parasuis* can affect the vascular endothelial barrier by downregulating vascular TJ expression and disrupting the distribution of TJ proteins (ZO-1, Occludin, Claudin-5, and JAM-1) [48]. It has been reported that *G. parasuis* could disrupt intercellular junctions and facilitate bacterial translocation across the tracheal epithelium by activating the p38/MAPK pathway [49]. In the current study, we found that the expression of tight junction proteins (ZO-1, Occludin, and Claudin-5) in the brains of mice decreased significantly after *G. parasuis* infection. Studies have reported that quercetin pretreatment could upregulate the mRNA expression of *Occludin* and *E-cadherin* to promote wound healing in atopic dermatitis (AD)-induced cells [50]. Therefore, quercetin may be used as an effective strategy for protecting the BBB integrity. In this study, we found that quercetin upregulated the expression levels of *ZO-1*, *Occludin*, and *Claudin-5* and maintained the intercellular junctions of ZO-1, Occludin, and Claudin-5 in mouse brain microvascular endothelial cells (bEnd.3). Therefore, we concluded that quercetin could protect BBB integrity during *G. parasuis* infection by enhancing TJ protein expression.

Matrix metalloproteinases (MMPs) are zinc-containing endopeptidases that digest extracellular matrix components that form the substrate surrounding the neurovascular unit [51]. In the acute-ischemic rat brain, MMP9 mediates the degradation of TJ proteins and angiogenesis in the BBB [52]. Vascular endothelial growth factor (VEGF) is associated with angiogenesis, neurogenesis, axonal plasticity, neuronal survival, and vascular permeability [53]. VEGF inhibition is an effective strategy for maintaining the integrity of the BBB in patients with acute ischemic stroke [54,55]. Early inhibition of VEGF may have a significant effect on cerebral ischemia, in part by regulating the expression of MMPs [52]. Angiopoietin-1 (Ang-1) and angiopoietin-2 (Ang-2) are secreted vascular growth factors that exert downstream signaling through Tie2 receptor tyrosine kinases [56]. Ang-1 has been reported to prevent blood vessel leakage [57], and Ang-2 has been shown to be a major regulator of BBB permeability [58]. Endothelin-1 (ET-1) is the most effective vasoconstrictor to date, and is involved in BBB dysfunction and brain edema [13]. ET-1 and MMPs-2, -9, and -14 in cerebrospinal fluid have been used as indicators of BBB dysfunction [59]. Therefore, in this study, we selected Mmp9, Vegf, Ang-1, Ang-2, and Et-1 as markers for BBB permeability. The expression levels of *Mmp9*, *Vegf*, *Ang-2*, and *Et-1* were significantly increased after *G. parasuis* infection both in vivo and in vitro. Quercetin can significantly decrease the *G. parasuis*-induced upregulation of *Mmp9*, *Vegf*, *Ang-2*, and *Et-1* in bEnd.3 cells, suggesting that quercetin alleviated the BBB permeability induced by *G. parasuis*.

Although we demonstrated the protective effects of quercetin on inflammatory responses and the integrity of the mouse brain microvascular endothelial cell monolayers in the *G. parasuis*-infected bEnd.3 cell model, the protective effect of quercetin in animal models has not been validated. Therefore, in the following study, we would validate the protective effects of quercetin in the *G. parasuis*-infected mouse model and add the inhibitors of the PI3K/Akt/Erk signaling pathway to explore the detailed mechanism associated with the PI3K/Akt/Erk signaling pathway. In accordance with the results in mice, we could also utilize the treatment of quercetin in the *G. parasuis*-infected pig model to demonstrate the possibility of quercetin as a new alternative substitute for preventing and controlling G. *parasuis* infection in pigs.

## 5. Conclusions

In conclusion, we demonstrated that quercetin had a protective effect on the BBB integrity during *G. parasuis* infection. Quercetin suppressed the expression of inflammatory cytokines (*Il-18*, *Il-6*, *Il-8*, and *Tnf-α*) and BBB-permeability marker genes (*Mmp9*, *Vegf*, *Ang-2*, and *Et-1*), promoted the expression of angiogenetic genes (*Sema4D* and *PlexinB1*), reduced *G. parasuis*-induced tight junction disruption, and reactivated *G. parasuis*-induced suppression of the PI3K/Akt/Erk signaling pathway in vitro. These findings shed light on the molecular mechanism by which quercetin exerts protective effects on the BBB integrity, and may provide a scientific basis for the application of quercetin as a dietary supplement for preventing and controlling bacterial meningitis.

## Figures and Tables

**Table 1:** Primers used for qRT-PCR.

Gene	Nucleotide Sequences (5′–3′)		Tm (°C)	Length (bp)
Il-1β	Forward	TGCCACCTTTTGACAGTGATG	59.04	138
	Reverse	TGATGTGCTGCTGCGAGATT	60.39	
Il-6	Forward	GCCCACCAAGAACGATAGTC	56.90	104
	Reverse	GTCGTTGTCACCAGCATCAG	56.60	
Il-8	Forward	GGCTTTGCGTTGATTCTGG	58.90	217
	Reverse	CGGTGTCCTGATTATCGTCCT	59.00	
Il-10	Forward	GCTGGACAACATACTGCTAACC	58.60	162
	Reverse	TCACCCAGGGAATTCAAATG	59.30	
Il-18	Forward	GGACACTTTCTTGCTTGCCA	60.00	166
	Reverse	CAGCCTCGGGTATTCTGTTATG	59.70	
Tnf-α	Forward	GCCCCCAGTCTGTATCCTTCTA	58.60	209
	Reverse	TTCGGAAAGCCCATTTGAGT	59.30	
Sema4D	Forward	CTGCCTCTTTTCCTACAACTGCT	60.00	212
	Reverse	GCTCCGTTTCATAGCCCGTA	60.40	
PlexinB1	Forward	CCCCTTCTAGAGCTTTCCGTG	60.80	121
	Reverse	GCAGCCTGGATAAGACCGTAA	59.60	
PI3K	Forward	GAAGATGATGAGGATTTGCCC	58.50	174
	Reverse	CTTGACTTCGCCGTCTACCAC	59.80	
Akt	Forward	CGGTTCTTTGCCAACATCGT	59.41	156
	Reverse	TAGGAGAACTTGATCAGGCGG	59.24	
Erk	Forward	CACCCATACCTGGAGCAGTA	58.50	221
	Reverse	TACACCATCTCTCCCTTGCTAT	58.07	
ZO-1	Forward	CAAAGGGAAAACCCGAAAC	57.20	184
	Reverse	GATACTGAGTTGCCTTCACCCT	57.70	
Occludin	Forward	CCCCTCTTTCCTTAGGCGAC	59.82	168
	Reverse	TTCAAAAGGCCTCACGGACA	59.82	
Claudin-5	Forward	GGGTGAGCATTCAGTCTTTAGC	58.50	174
	Reverse	ACAGCCCCTTCCAAGTCGT	59.10	
Vegf	Forward	GCACTGGACCCTGGCTTTAC	60.96	144
	Reverse	GTCTCAATCGGACGGCAGTA	59.55	
Mmp9	Forward	TCTAGGCCCAGAGGTAACCC	60.03	141
	Reverse	TTATCCACGCGAATGACGCT	60.18	
Ang-1	Forward	CTGAAGAGTTGACACAGGGCT	59.93	119
	Reverse	ATCTGGGCCATCTCCGACTT	60.69	
Ang-2	Forward	CCGCGGGCAAAATAAGTAGC	59.97	126
	Reverse	CACATGCGTCAAACCACCAG	60.04	
Et-1	Forward	GGCCCAAAGTACCATGCAGA	60.32	137
	Reverse	TGCTATTGCTGATGGCCTCC	60.18	
Gapdh	Forward	AAGCCCATCACCATCTTCCA	60.00	88
	Reverse	CACCAGTAGACTCCACGACA	60.00	

## Data Availability

All relevant data have been presented as an integral part of this manuscript. The data that support the findings of this study are available from the corresponding authors upon reasonable request.

## Supplementary Materials

The following supporting information can be downloaded at: https://www.mdpi.com/article/10.3390/biom14060696/s1, Figure S1: H&E staining of mouse cerebral tissue after 24, 48, and 72 h infection with GPS. Original western blots are available in the Supplementary Materials.
