# Supplementary material for: Quercetin Protects Blood–Brain Barrier Integrity via the PI3K/Akt/Erk Signaling Pathway in a Mouse Model of Meningitis Induced by Glaesserella parasuis
Source: Biomolecules. 2024 Jun 14;14(6):696. doi: 10.3390/biom14060696 (PMC11201931; doi:10.3390/biom14060696)

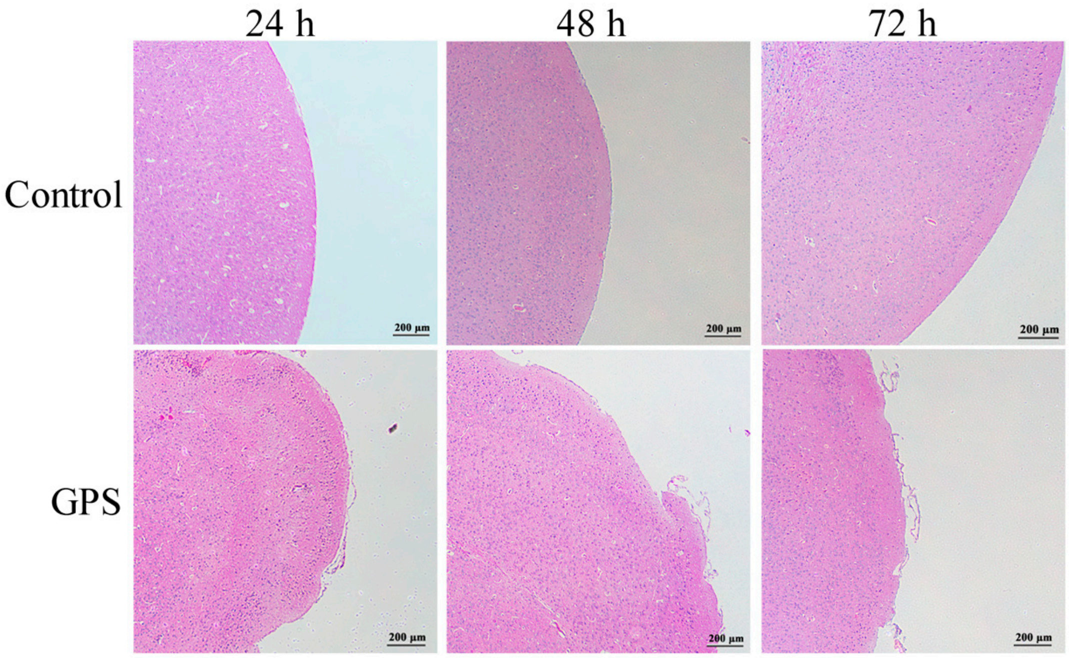

Figure S1. H&E staining of mouse cerebral tissue after 24, 48, and 72 h infection with GPS (magnification 10 × 10).

Original Western blots pictures:

Figure 3

Figure 3B

Sema4D (repeat 1, repeat 2, repeat 3)

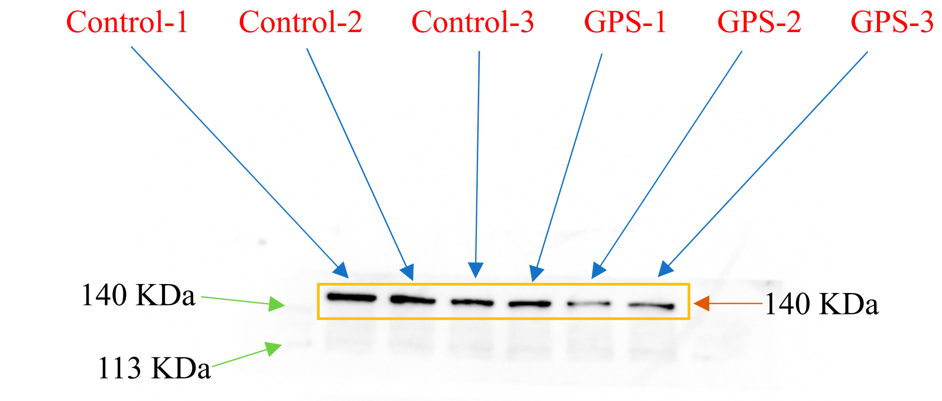

Sema4D (repeat 4, repeat 5, repeat 6)

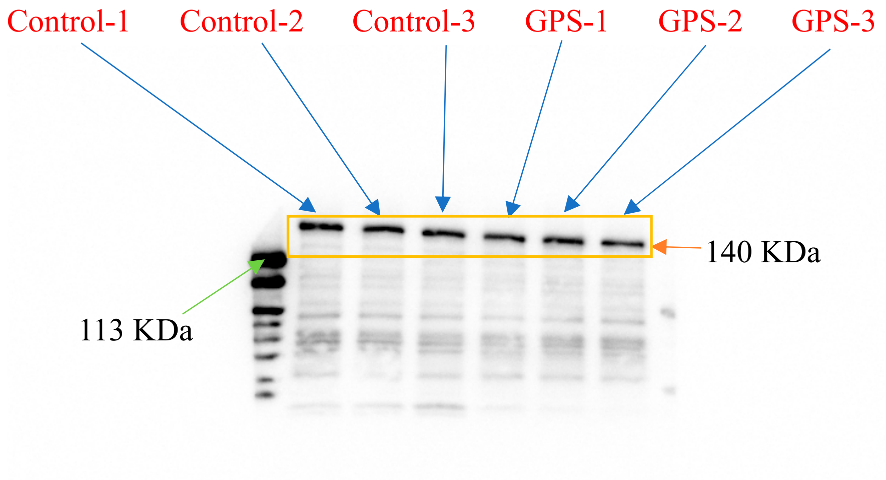

PlexinB1 (repeat 1, repeat 2, repeat 3)

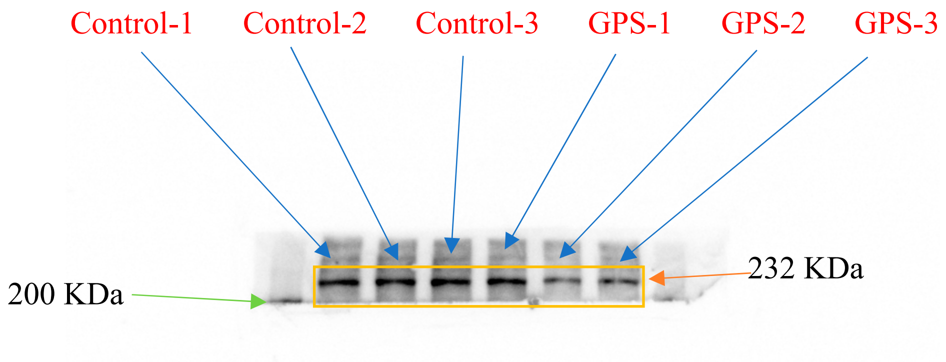

PlexinB1 (repeat 4, repeat 5, repeat 6)

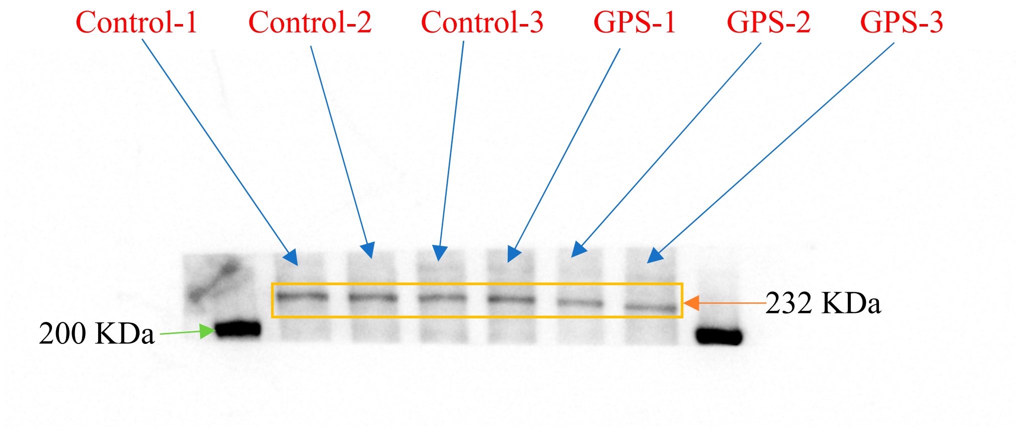

GAPDH (repeat 1, repeat 2, repeat 3)

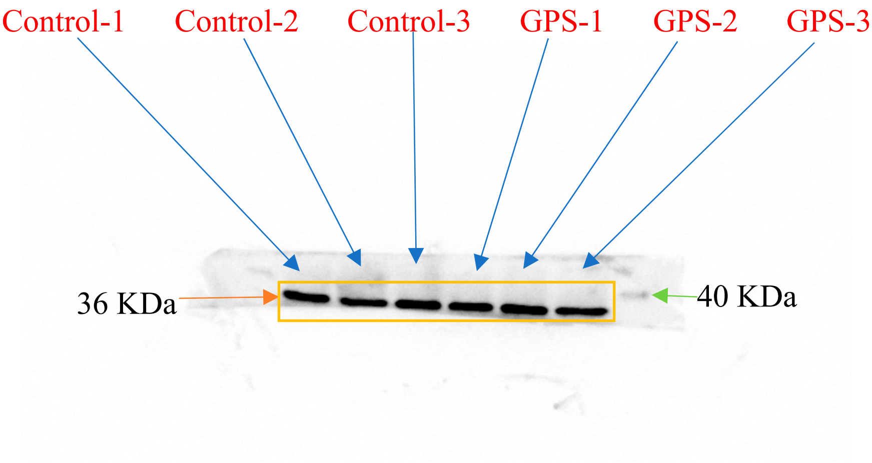

GAPDH (repeat 4, repeat 5, repeat 6)

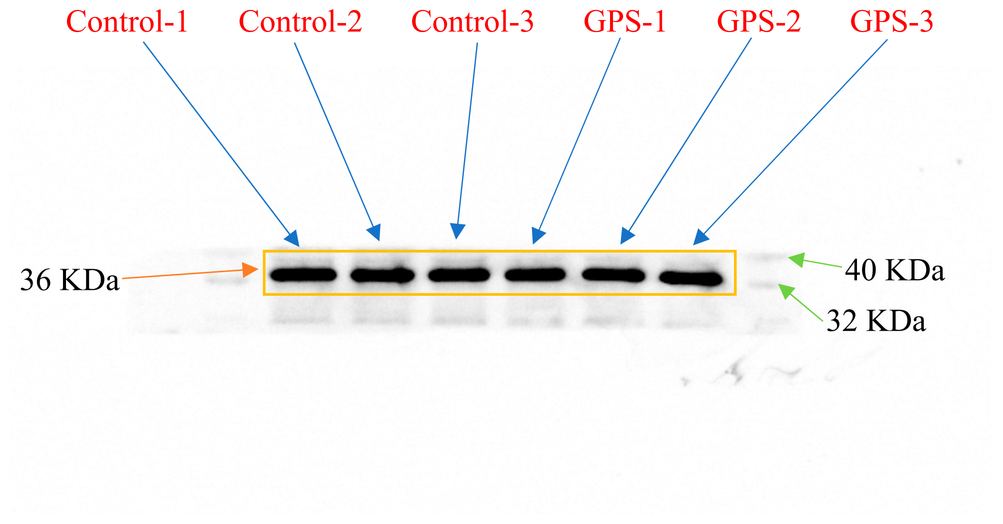

Figure 6

Figure 6B

PI3K (repeat 1, repeat 2, repeat 3)

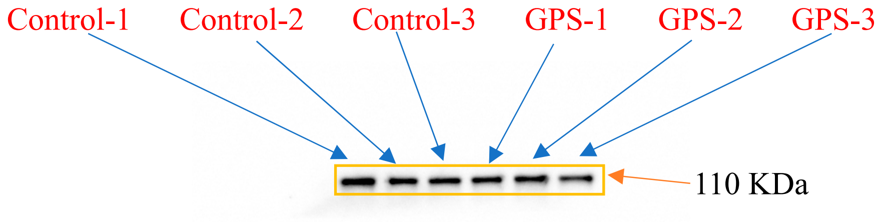

PI3K (repeat 4, repeat 5, repeat 6)

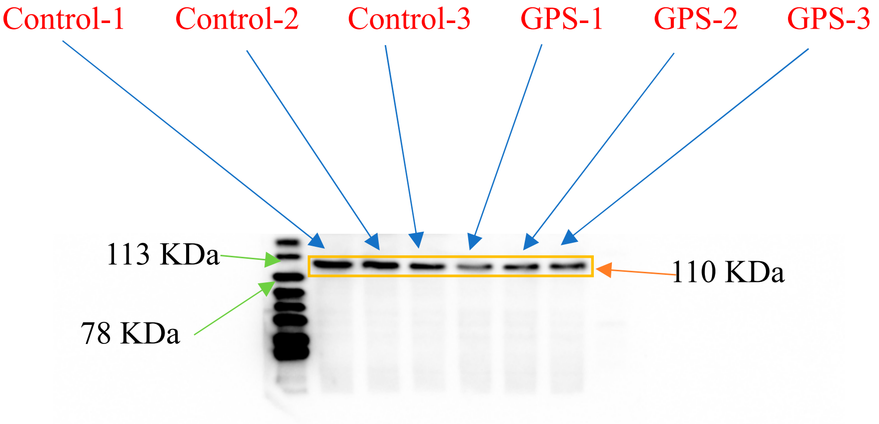

p-PI3K (repeat 1, repeat 2, repeat 3)

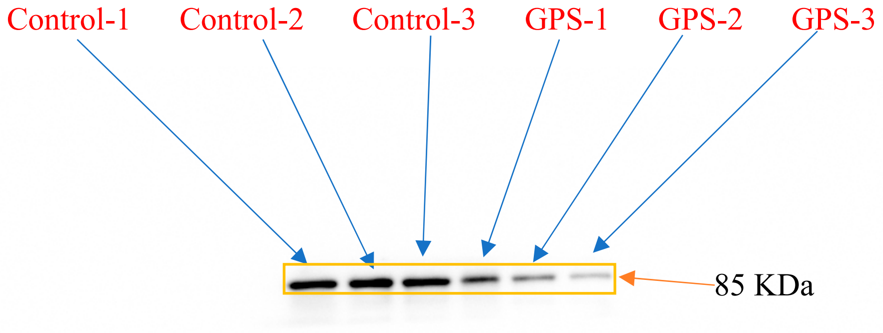

p-PI3K (repeat 4, repeat 5, repeat 6)

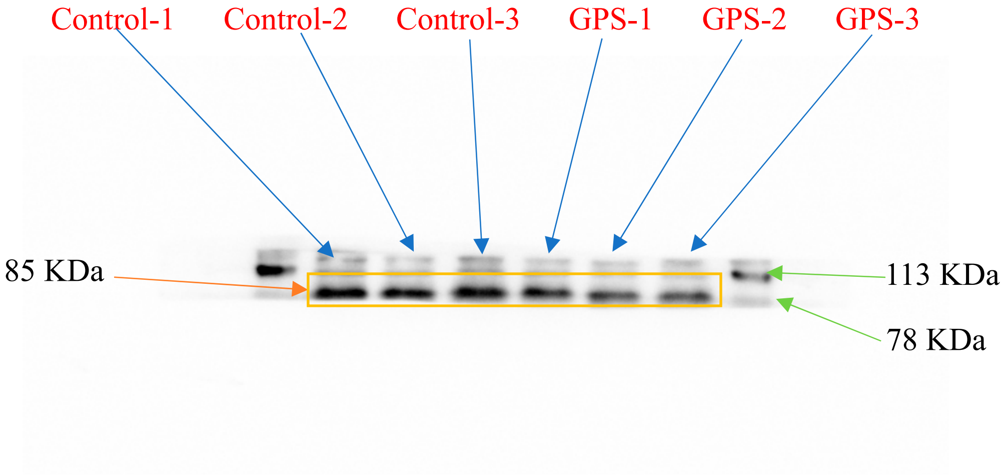

AKT (repeat 1, repeat 2, repeat 3)

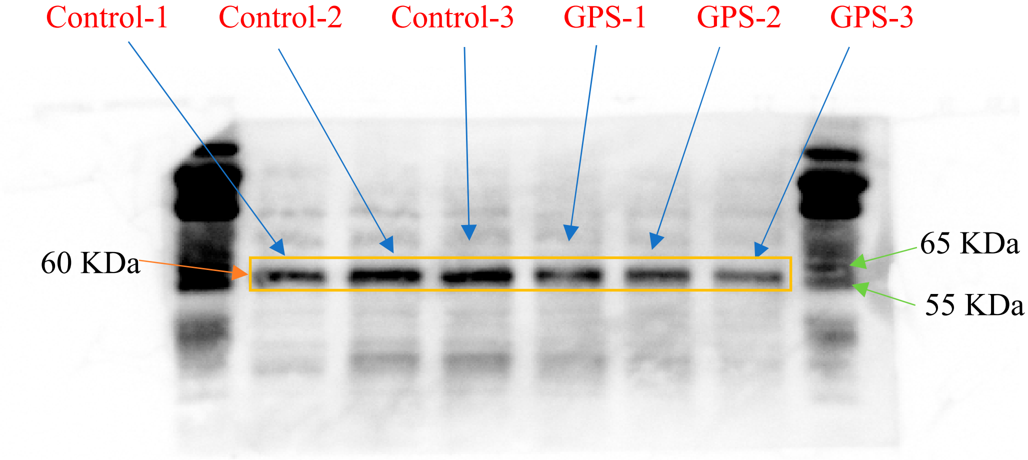

p-AKT (repeat 1, repeat 2, repeat 3)

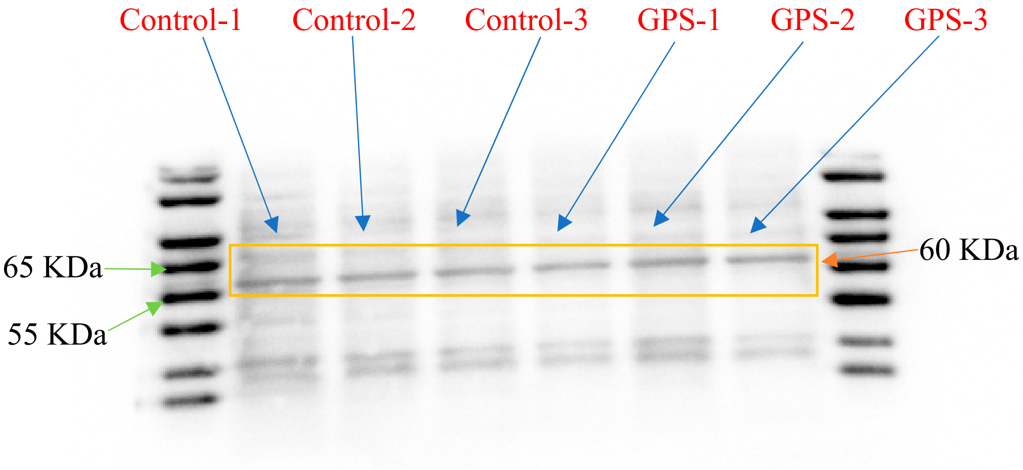

ERK (repeat 1, repeat 2, repeat 3)

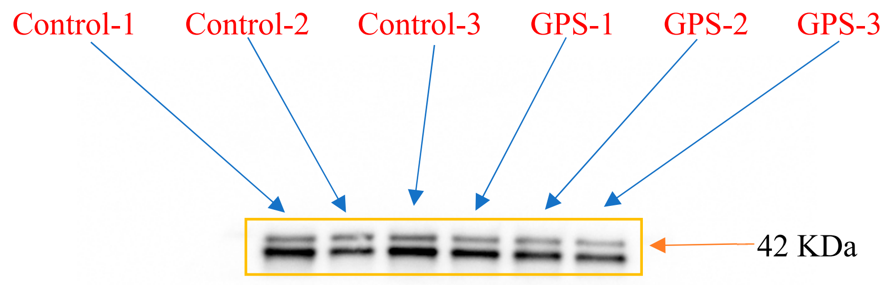

ERK (repeat 4, repeat 5, repeat 6)

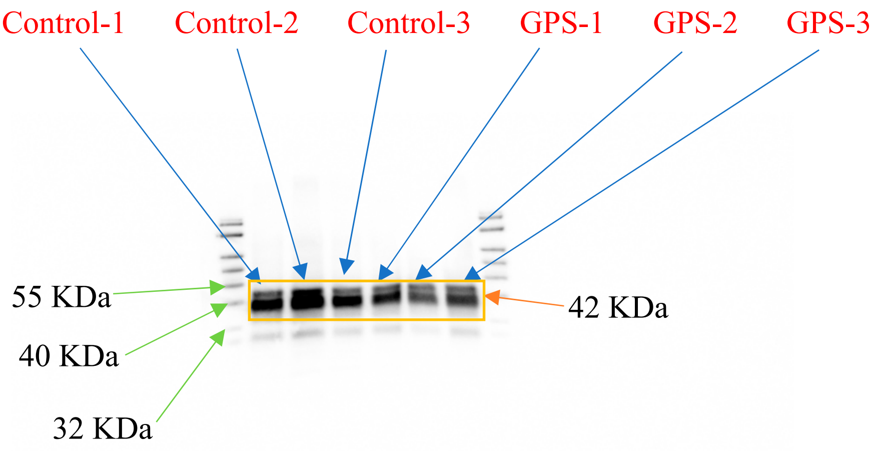

p-ERK (repeat 1, repeat 2, repeat 3)

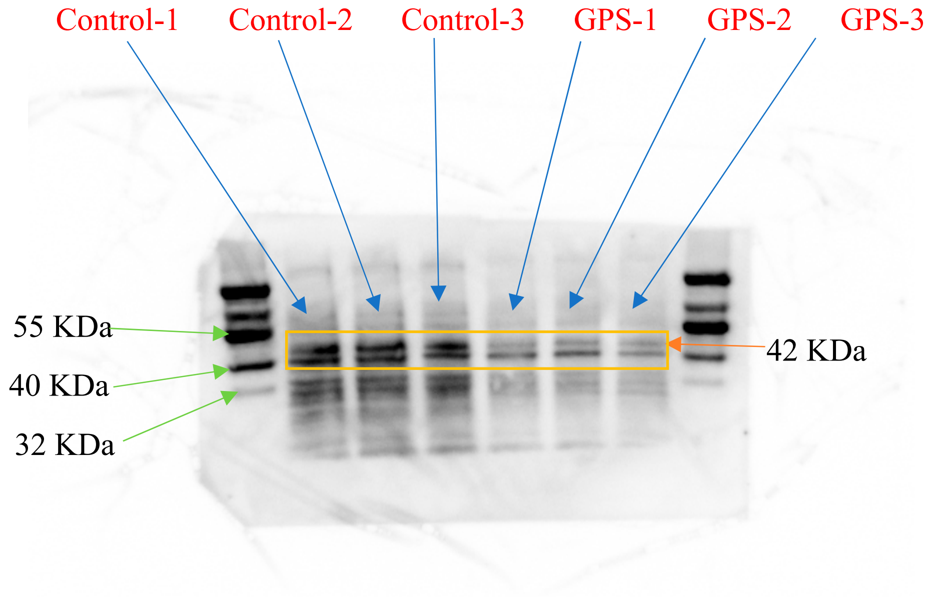

GAPDH (repeat 1, repeat 2, repeat 3)

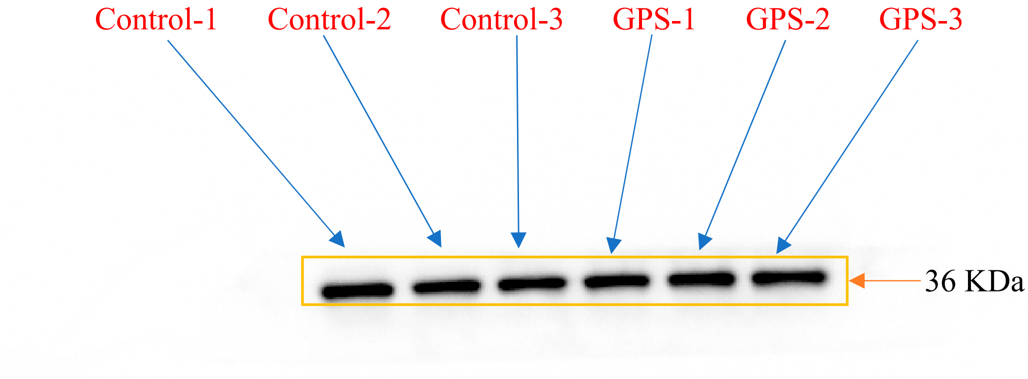

GAPDH (repeat 4, repeat 5, repeat 6)

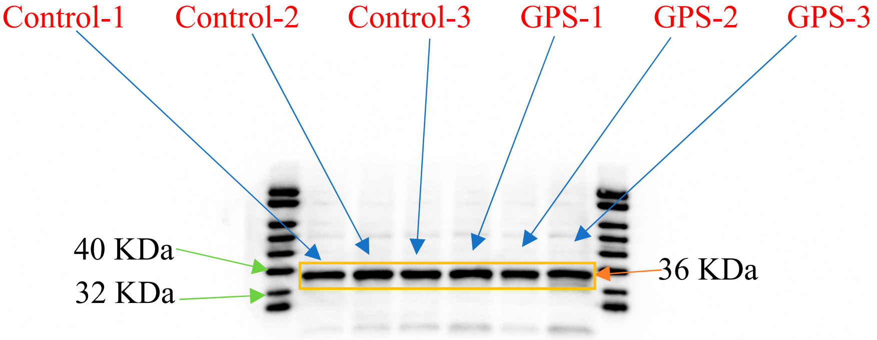

Figure 11

Figure 11B

PI3K (repeat 1)

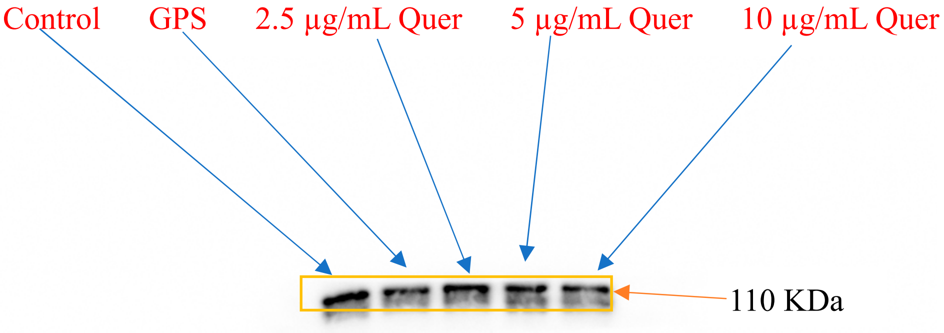

PI3K (repeat 2)

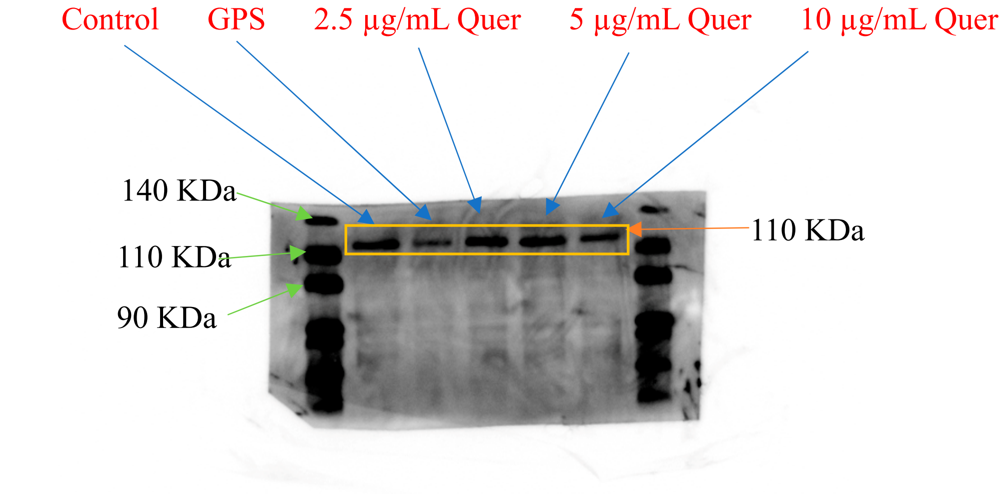

PI3K (repeat 3)

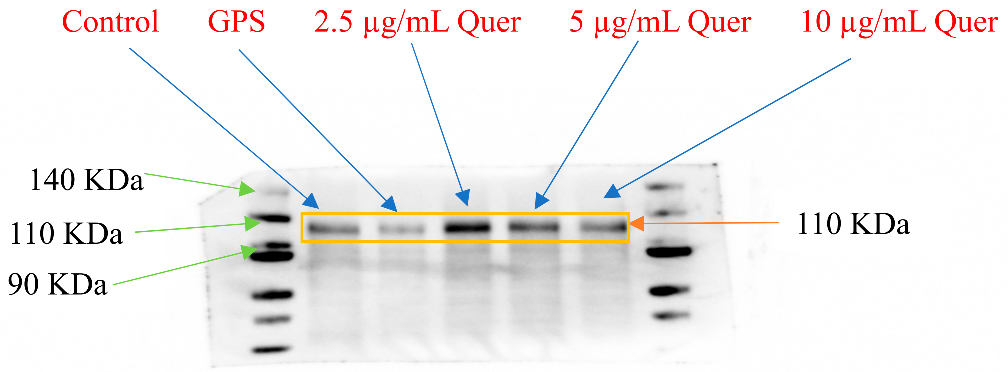

PI3K (repeat 4)

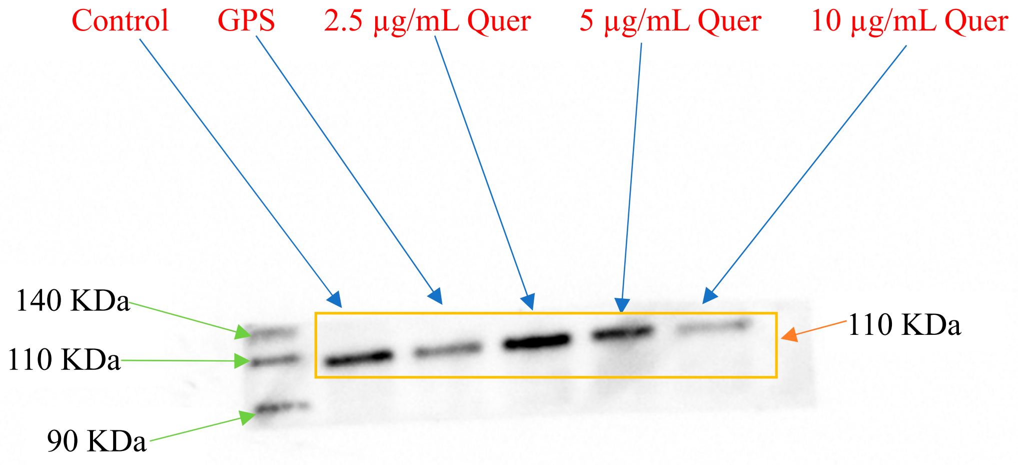

p-PI3K (repeat 1)

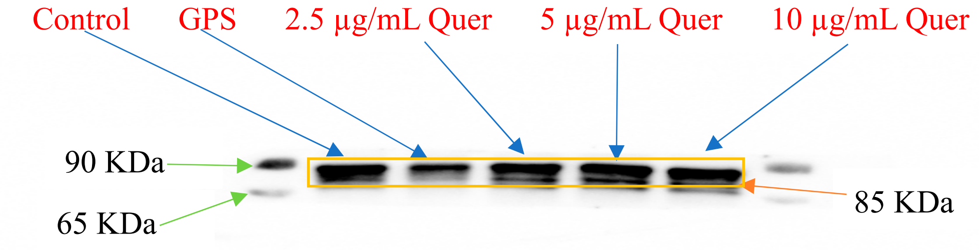

p-PI3K (repeat 2)

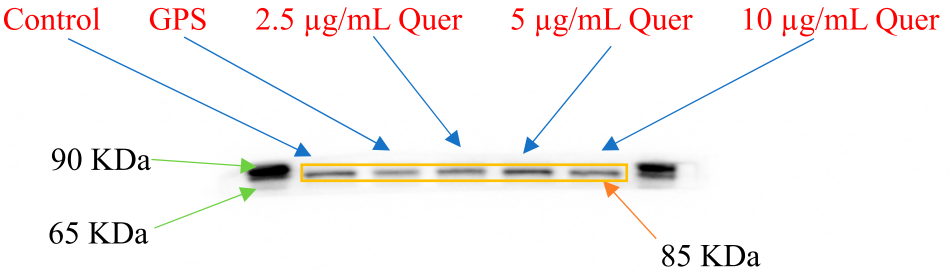

p-PI3K (repeat 3)

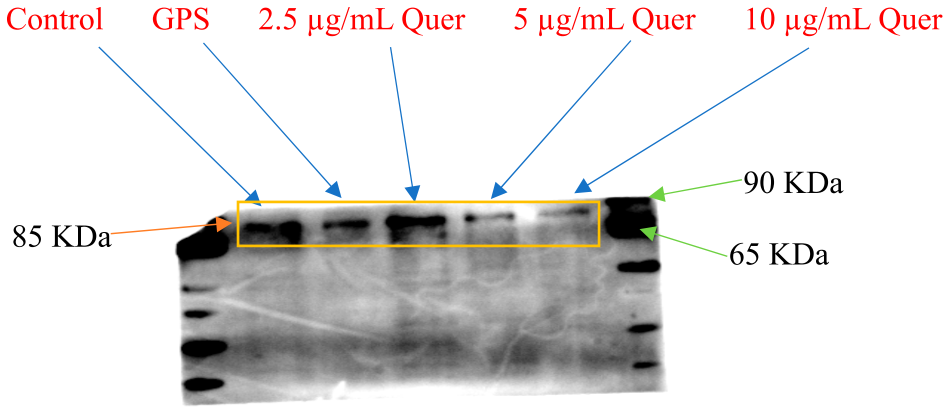

AKT (repeat 1)

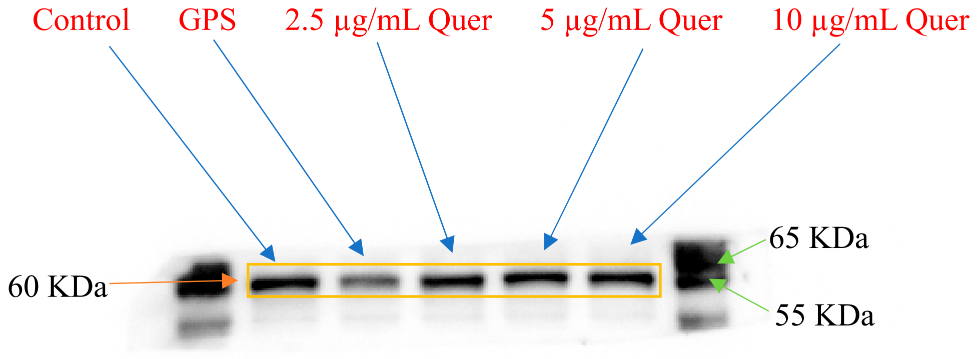

AKT (repeat 2)

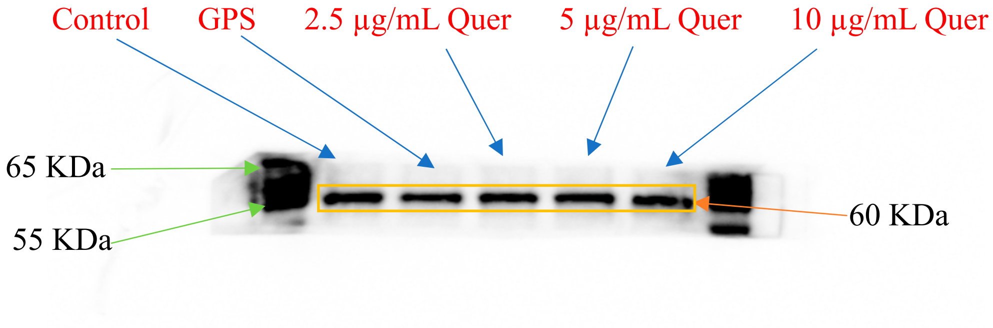

AKT (repeat 3)

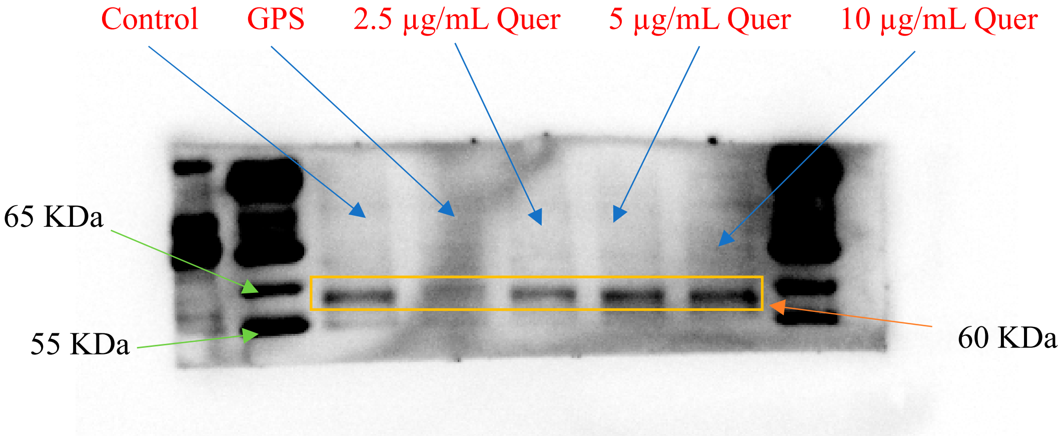

p-AKT (repeat 1)

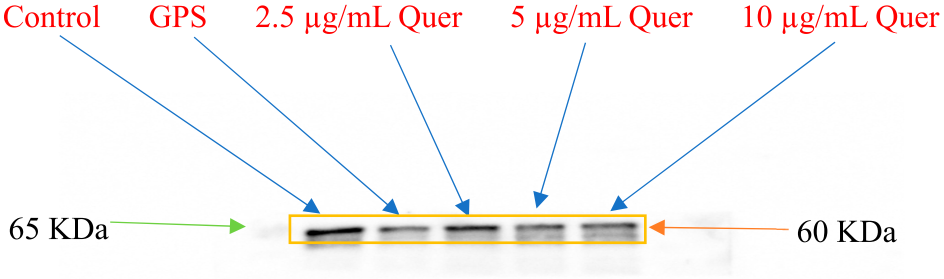

p-AKT (repeat 2)

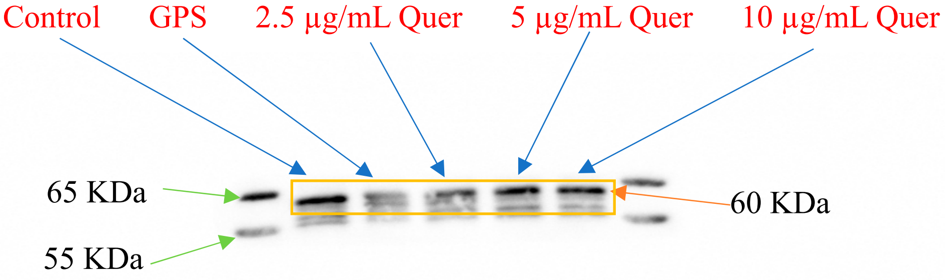

p-AKT (repeat 3)

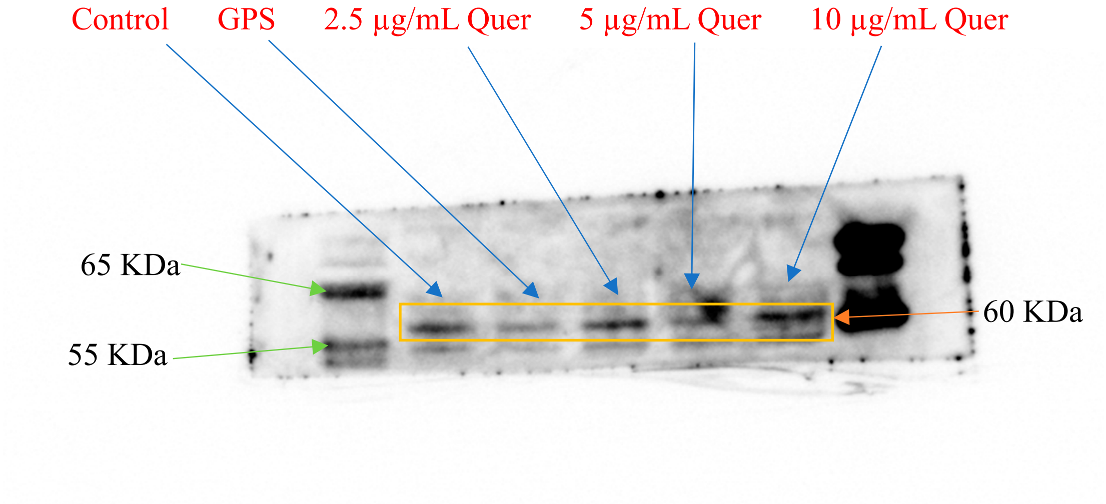

p-AKT (repeat 4)

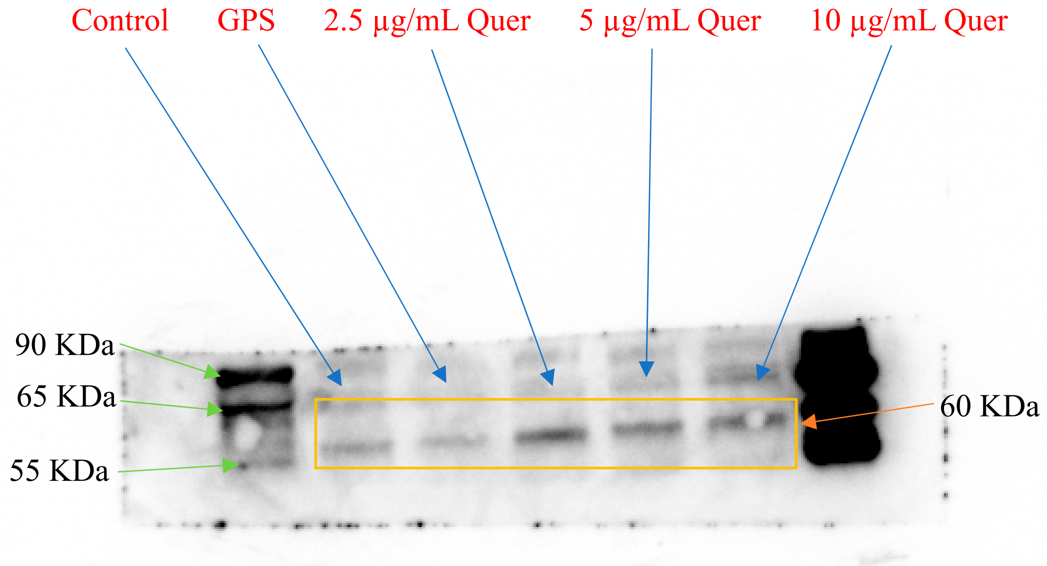

p-AKT (repeat 5)

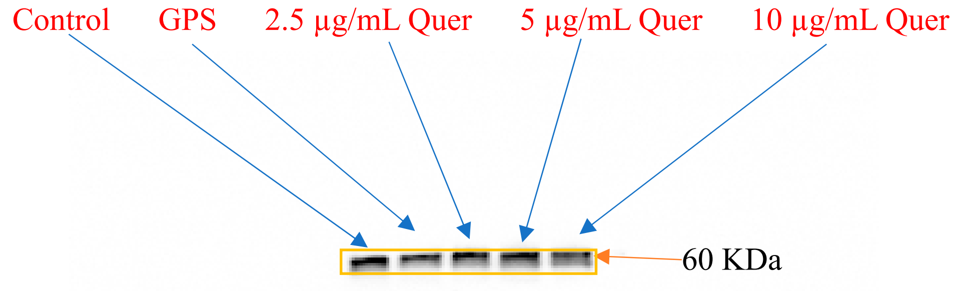

ERK (repeat 1)

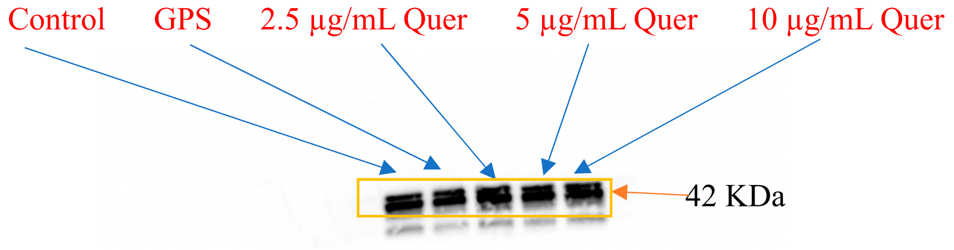

ERK (repeat 2)

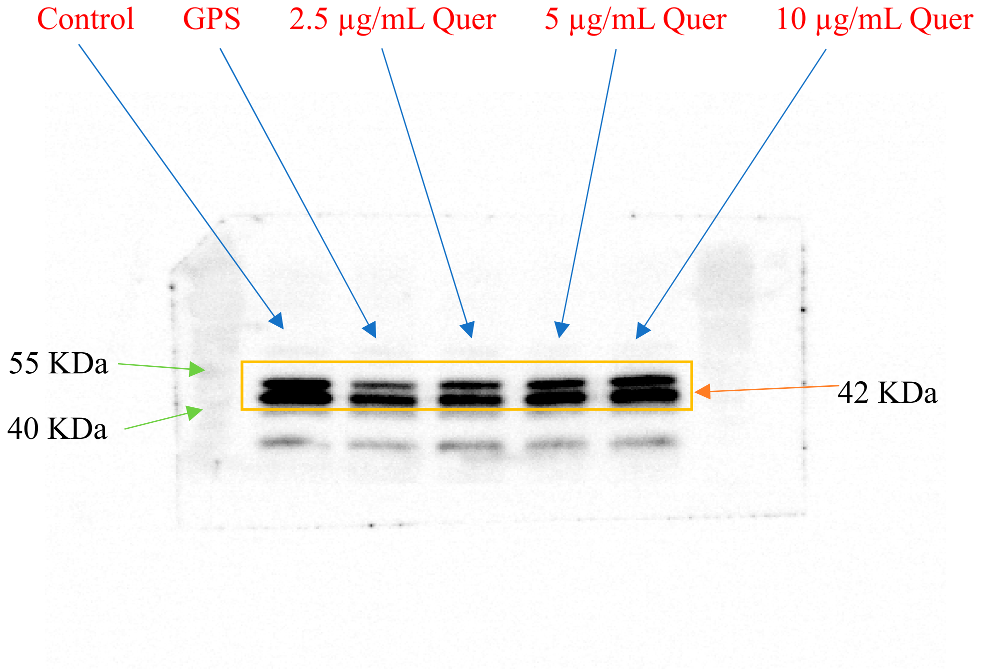

ERK (repeat 3)

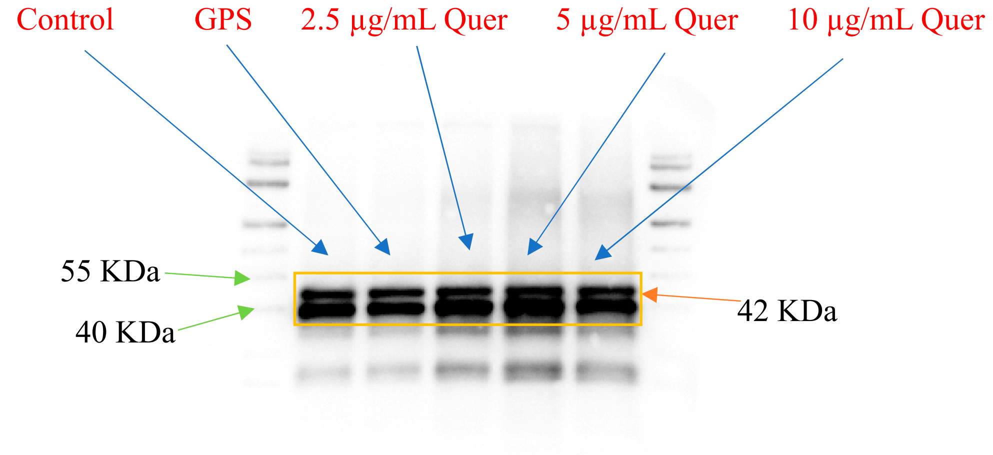

ERK (repeat 4)

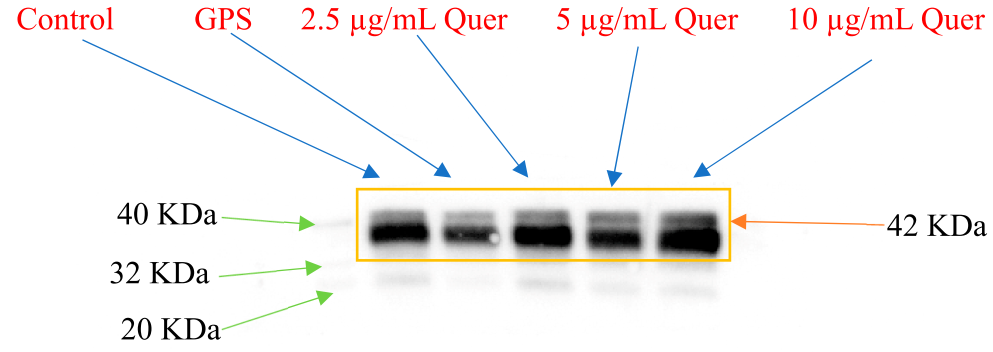

p-ERK (repeat 1)

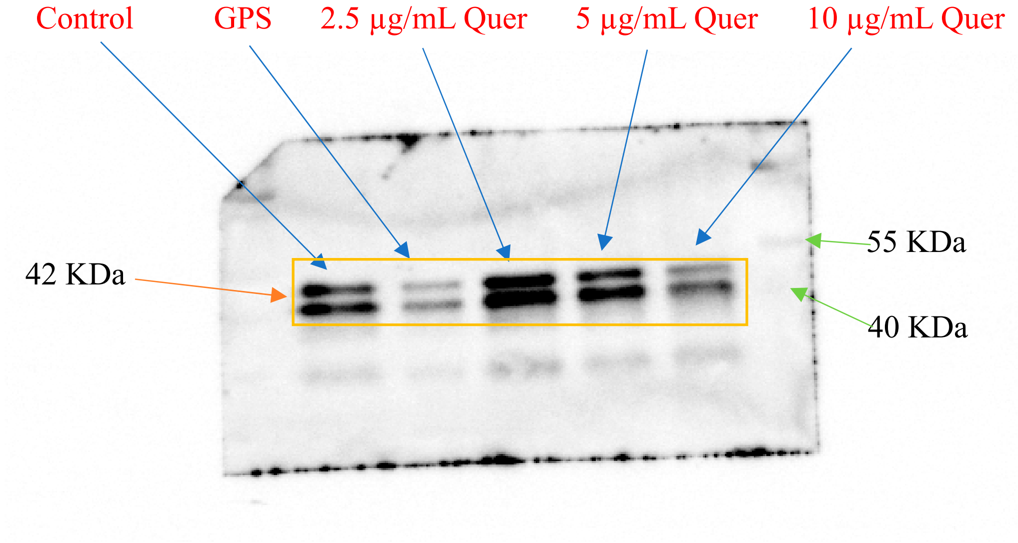

p-ERK (repeat 2)

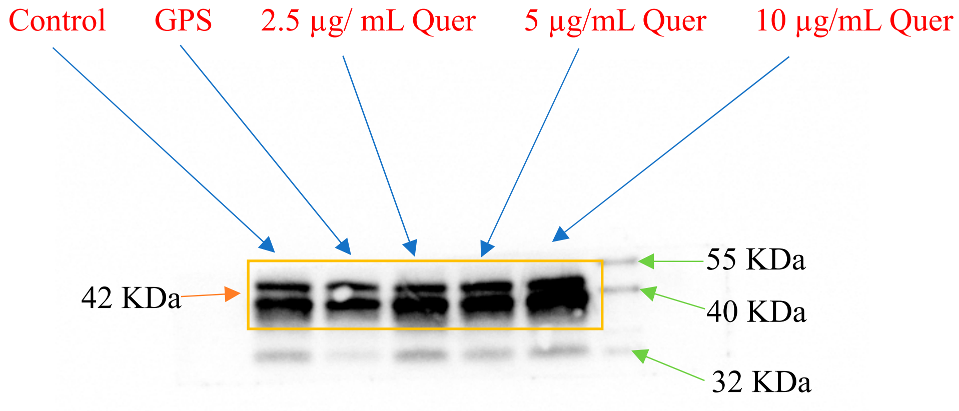

p-ERK (repeat 3)

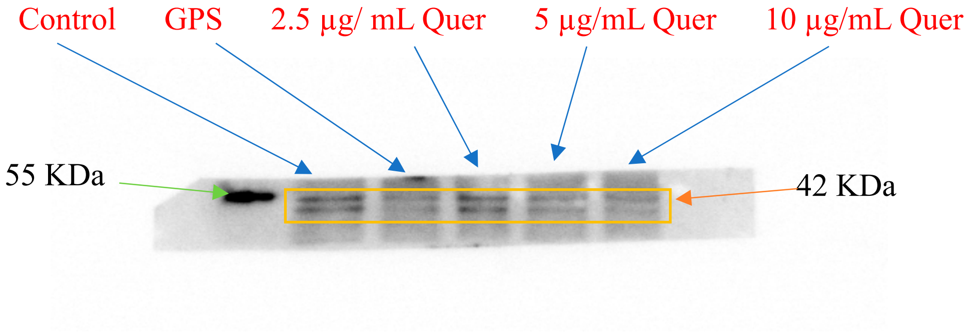

p-ERK (repeat 4)

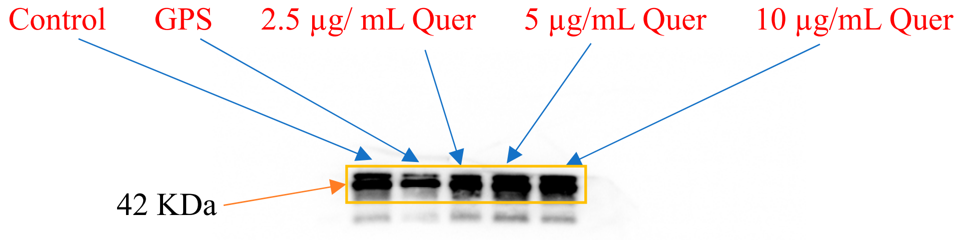

GAPDH (repeat 1)

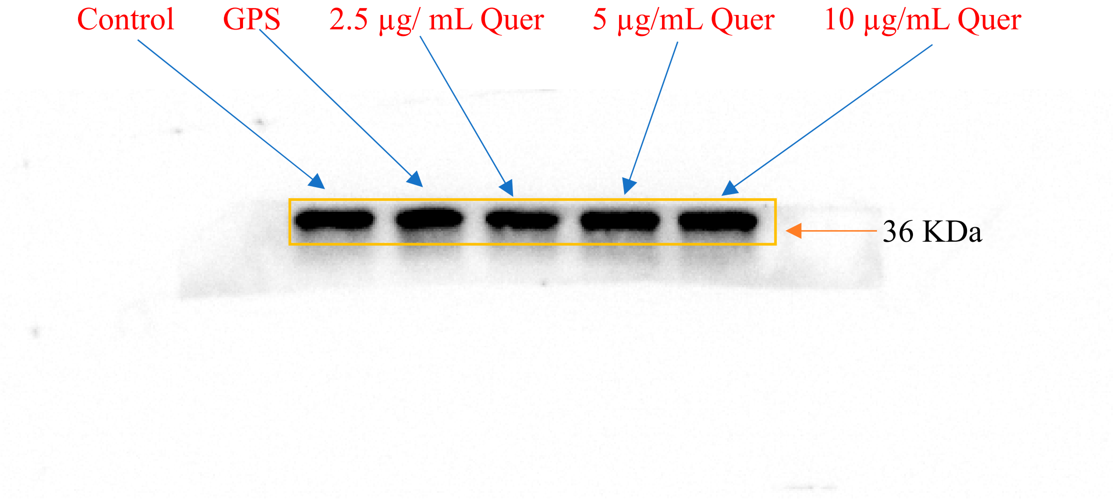

GAPDH (repeat 2)

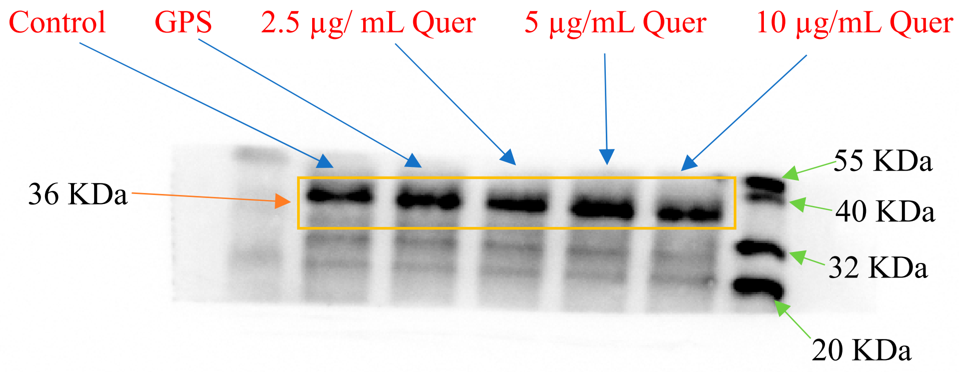

GAPDH (repeat 3)

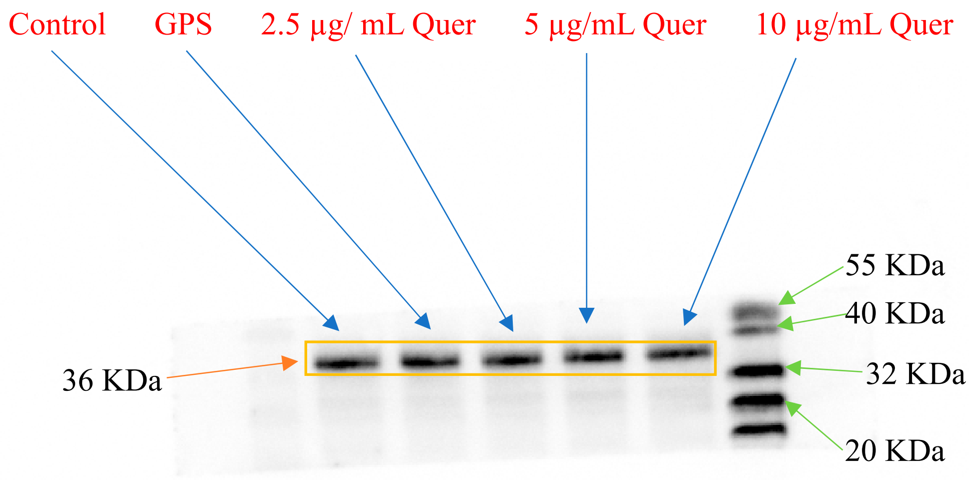

GAPDH (repeat 4)

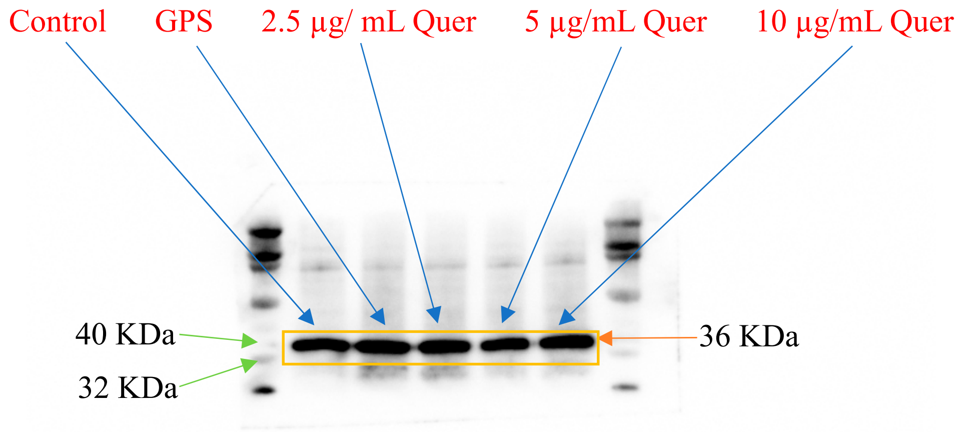

Supplement: Supplementary file 1 [file biomolecules-14-00696-s001.zip › biomolecules-3025892-supplementary.pdf]
